# Complementary and alternative metrics for tracking population-level trends in child linear growth

**DOI:** 10.1371/journal.pgph.0001766

**Published:** 2023-04-17

**Authors:** Ashley M. Aimone, Diego G. Bassani, Huma Qamar, Alison Dasiewicz, Nandita Perumal, Sorrel M. L. Namaste, Devanshi Shah, Daniel E. Roth

**Affiliations:** 1 Centre for Global Child Health, The Hospital for Sick Children, Toronto, Ontario, Canada; 2 Department of Paediatrics, The Hospital for Sick Children and University of Toronto, Toronto, Ontario, Canada; 3 Dalla Lana School of Public Health, University of Toronto, Toronto, Ontario, Canada; 4 Department of Global Health and Population, Harvard TH Chan School of Public Health, Boston, Massachusetts, United States of America; 5 The DHS Program, ICF, Rockville, Maryland, United States of America; Wageningen University & Research, NETHERLANDS

## Abstract

Stunting prevalence is commonly used to track population-level child nutritional status. However, other metrics derived from anthropometric datasets may be used as alternatives to stunting or provide complementary perspectives on the status of linear growth faltering in low- and middle-income countries (LMICs). Data from 156 Demographic and Health Surveys in 63 LMICs (years 2000 to 2020) were used to generate 2 types of linear growth metrics: (i) measures of location of height distributions (including stunting) for under-5 years (<5y) and 2 to 5 years (2-5y); (ii) model-derived metrics including predicted mean height-for-age z-score (HAZ) at 0, 2, and 5 years; interval slopes of HAZ, height-for-age difference (HAD), and growth delay (GD) from 1 month to 2 years (1mo-2y) and 2-5y; and the SITAR intensity parameter (SITAR-IP) for <5y. Using Spearman’s rank correlation coefficient (*r*), metrics were considered alternatives to stunting if very strongly correlated with stunting (|*r*|≥0.95) and at least as strongly correlated as stunting with selected population indicators (under 5y mortality, gross domestic product, maternal education). Metrics were considered complementary if less strongly correlated with stunting (|*r*|<0.95) yet correlated with population indicators. We identified 6 of 15 candidate metrics (stunting 2-5y, mean HAZ <5y and 2-5y, p25 HAZ <5y and 2-5y, predicted HAZ at 2y) as potential alternatives to stunting and 6 as complementary metrics (SITAR-IP, predicted HAZ at 5y, HAZ slope 1m-2y, HAD slope 1m-2y, GD slopes 1m-2y and 2-5y). Three metrics (HAZ slope 2-5y, HAD slope 2-5y years and predicted HAZ at birth) had weak correlations with population indicators (|*r*| ≤ 0.43). In conclusion, several linear growth metrics could serve as alternatives to stunting prevalence and others may be complementary to stunting in tracking global progress in child health and nutrition. Further research is needed to explore the real-world utility of these alternative and complementary metrics.

## Introduction

Reducing the burden of child growth faltering is a global public health priority represented within the United Nations Sustainable Development Goals (SDGs) [1]. Population-level metrics based on child height data are indicators of childhood nutritional status and are associated with economic and social development [2]. Stunting prevalence, defined as the proportion of children in a population with a height-for-age z-score (HAZ) more than 2 standard deviations below the median of the World Health Organization (WHO) Child Growth Standard [3], is a conventional metric of population-level linear growth faltering that is widely used to track trends in childhood nutritional status and measure the effectiveness of related public health programming in low- and middle-income countries (LMICs) [4].

Stunting prevalence is straightforward to estimate, does not rely on an assumption that a given height distribution is normal, and is established in programmatic and policy circles [5]. However, the z-score cut-point (HAZ < -2) that defines stunting does not have a clinical or biological basis, and the common use of stunting often incorrectly implies that the burden of linear growth faltering only affects a ‘stunted’ subgroup of the population while ‘non-stunted’ children have normal growth [6, 7]. In fact, linear growth faltering in most LMICs is a whole-population phenomenon resulting from a shift in the entire height distribution, such that even the tallest children in many LMICs are generally shorter than they would have been under optimal conditions for growth [6]. Given that stunting is based on the lower tail of the height distribution, it may not represent the whole-population shift and may be more susceptible to differential errors or imprecision in measurements as compared to measures of central tendency (e.g., mean). Furthermore, the prevalence of stunting <5y is based on all children from birth to 5 years, whereas other metrics derivable from height-age survey data capture the dynamics of the height trajectory across defined age ranges.

In this study, we considered a range of height distribution-based child linear growth metrics derived from anthropometric survey data which may have statistical and/or conceptual advantages over stunting prevalence and therefore be potentially useful as alternative or complementary metrics (i.e., such metrics could be used in place or alongside the conventional indicator of stunting prevalence <5y). Building on prior work summarized in a Demographic and Health Survey (DHS) working paper [8], the specific aims of this study were to assess candidate linear growth metrics based on the strength of their correlations with stunting prevalence and to compare the validity of these candidate linear growth metrics based on the strength of correlations with three key indicators of population health and development: under 5y mortality, gross domestic product (GDP), and maternal education.

## Methods

### Data source

Data used for this study were from The DHS Program, a primary source of population-representative anthropometric data for LMICs. The data collection methodology for The DHS Program is described in detail elsewhere [9, 10]. Individual-level height, sex, date of birth, and date of interview data were obtained from 156 phase IV and above DHS surveys for 63 LMICs between the years 2000 and 2020 (S1 Table). HAZs were generated using the WHO Child Growth Standards (WHO-GS) [3]. Individual child data were excluded from the analysis if month or year of birth was missing, if the child had not slept in the household the previous night (not a de facto resident), or if their height measurement was flagged as implausible (HAZ <-6 or >6 based on WHO-GS criteria). If day of birth was missing, day 15 (midpoint of the month) was imputed to calculate the child’s age [11]. Whole surveys were excluded from the analysis if data were collected only on children of female respondents, if the anthropometric data were suppressed from the DHS final report due to quality issues), or if data were unavailable for all three population indicators (as described below).

This study is a secondary analysis of data that are publicly available from The DHS Program and was therefore exempt from ethics review. Ethical approval for conducting the surveys was provided by each host country and the ICF Institutional Review Board (IRB). Ethics approval documentation for any survey may be obtained from The DHS Program (https://dhsprogram.com/Methodology/Request-for-documentation-of-ethical-review.cfm).

### Linear growth metric selection and calculation

We selected several child linear growth metrics used in a previous analysis [8] including those based on HAZ and height-for-age difference (HAD) [12]. We prioritized measures of central tendency and slopes rather than metrics based on cut-points in the tails of the height distributions. Compared to the prior study [8], we removed some candidate metrics (e.g., median HAZ) to reduce unnecessary redundancy (e.g., median and mean HAZ are highly correlated). We added new metrics based on growth delay (GD), which we recently proposed to express linear growth faltering as a delay in skeletal maturation on the time scale (also referred to as growth tempo) rather than a deficit in stature [13]. We also included the Super-Imposition by Translation and Rotation (SITAR) intensity parameter [14], which is a scaling factor that reflects the velocity of a linear growth curve relative to the mean velocity. The SITAR size parameter was not included as we previously found it to be nearly interchangeable with <5y mean HAZ [15]. Metrics reported in the literature that incorporate stunting prevalence rather than present new statistical expressions of height-age distributions (e.g., “Composite Index of Anthropometric Failure”) were not considered [16]. We refer to statistical expressions of linear growth as ‘metrics’ to avoid inconsistencies with common uses of the terms ‘measurements’, ‘indices’, and ‘indicators’ in the nutritional epidemiology literature [7, 17].

**Fig 1:**
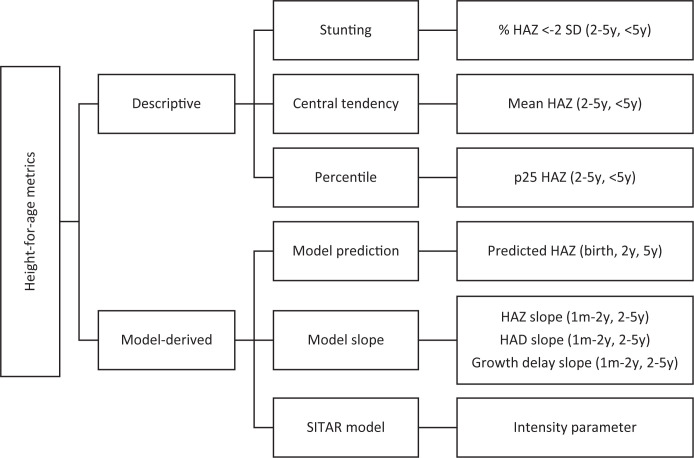
Descriptive and model-derived linear growth metrics.

Using individual child-level data, two types of linear growth metrics were generated for each survey (Fig 1; Table 1). Descriptive metrics were estimated directly from the observed height/HAZ distribution and divided into 3 classes: (i) stunting prevalence (reported as a proportion), (ii) measures of central tendency, and (iii) percentiles. Each descriptive metric was estimated for all children under the age of 5 years (<5y) and the 2 to 5 years (2-5y) age range. Given the population-average growth faltering that is common in LMICs up to 2 years of age (irrespective of the metric used) [13], cross-sectional metrics (i.e., mean, 25^th^ percentile, stunting) were not estimated for the period defined as 1 month to 2 years of age (1mo-2y).

**Table 1:** Definitions of linear growth metrics.

Linear growth metric	Definition and statistical notes
Mean Stunting Prevalence	Proportion of children within a specified age interval (less than 5 years of age, 2–5 years of age) with a height more than 2 standard deviations below the reference median for age and sex (HAZ <-2):
	$\frac{\left[\begin{array}{c} Number\;of\;children\;in\;age\;interval\\ with\;HAZ<‐2 \end{array}\right]}{\left[\mathrm{Number}\;\mathrm{of}\;\mathrm{children}\;\mathrm{in}\;\mathrm{age}\;\mathrm{interval}\right]}\times 100$
Mean HAZ	Sum of all individual child HAZ values in a specified age internal (less than 5 years of age, 2–5 years of age) divided by the number of values:
	$\frac{\sum \left({HAZ}_{1}+{HAZ}_{2}+\cdots {HAZ}_{n}\right)}{n}$
25^th^ Percentile HAZ	Value at the 25^th^ percentile of the observed HAZ distribution in a specified age interval (less than 5 years of age, 2–5 years of age).
Predicted HAZ	Predicted (mean) HAZ at a specified age (at birth, at 2 years of age, at 5 years of age) based on a linear spline regression model of HAZ versus age from birth to 5 years of age, with one knot at 731 days of age:
	$HAZ(age)=\beta_{1}({age}_{a})(age)+\beta_{2}({age}_{b})(age-731)+c$
	Where *age* is the observed age of a child, *age*_*a*_ and *age*_*b*_ are indicators for whether age is <731 days or ≥731 days, respectively, and *c* is the intercept.
	Although expressed in units of HAZ in the present analyses, these metrics could be re-expressed on either HAD or GD scales and would retain the same interpretation (i.e., mean HAZ, mean HAD and mean GD at one age timepoint are all nearly perfectly correlated with one another [13].
Slope of HAZ	Model-derived slope within a specified age interval (1 month-2 years of age, 2–5 years of age):
	${HAZ\;slope}_{{age}_{1}\;to\;{age}_{2}}=\frac{HAZ({age}_{1})-HAZ({age}_{0})}{{age}_{1}-{age}_{0}}$
	Where *HAZ* is the predicted HAZ based on the regression model described above, *age*_*0*_ is the age at the beginning of the interval, and *age*_*1*_ is the age at end of the interval.
	*Conventional interpretation*: A negative slope indicates further growth faltering, a positive slope denotes catch-up growth.
Slope of HAD	Height-for-age difference (HAD) is the difference between an observed height and the median age-specific height from the WHO growth standards. Here, predicted HAD at a specified age was derived from the predicted HAZ as follows (steps 1 and 2):
	Step 1: Length(age)=m×(HAZ(age)×s+1)
	Step 2: HAD(age)=Length(age)−m
	Where *m* is the average of the age-specific WHO-GS m parameters for boys and girls, *HAZ* is the predicted HAZ (see model above for ‘Predicted HAZ’), and *s* is the average of the age-specific WHO-GS s parameters for boys and girls [18]. The model-derived slope of HAD was then calculated within a specified age interval (1 month-2 years of age, 2–5 years of age):
	Step 3:
	${HAD\;slope}_{{age}_{0}\;to\;{age}_{1}}=\frac{HAD({age}_{1})-HAD({age}_{0})}{{age}_{1}-{age}_{0}}$
	Where *HAD* is the predicted HAD calculated in step 2, *age*_*0*_ is the age at the beginning of the interval, and *age*_*1*_ is the age at end of the interval.
	*Conventional interpretation*: A negative slope indicates further growth faltering; a positive slope denotes catch-up growth.
Slope of GD	Growth delay (GD) is the difference between the height-age and chronological age, calculated as follows (steps 1–3):
	Step 1: Length(age)=m×(HAZ(age)×s+1)
	Where *m* is the average of the age-specific WHO-GS m parameters for boys and girls, *HAZ* is the predicted HAZ (see model above for ‘Predicted HAZ’), and *s* is the average of the age-specific WHO-GS s parameters for boys and girls [18].
	Step 2: Determine the age corresponding to *Length(age)* in the WHO-GS [3].
	Step 3: $GD(age)=age-{age}_{WHO-GS}$
	Then, the model-derived slope of GD is calculated within a specified age interval (1 month-2 years of age, 2–5 years of age) [13].
	Step 4:
	${GD\;slope}_{{age}_{0}\;to\;{age}_{1}}=\frac{GD({age}_{1})-GD({age}_{0})}{{age}_{1}-{age}_{0}}$
	Where *HAZ* is the predicted HAZ, *age*_*0*_ is the age at the beginning of the interval, and *age*_*1*_ is the age at end of the interval.
	*Proposed interpretation*: A flat slope indicates complete catch-up growth; a positive slope indicates continued growth faltering.
SITAR intensity parameter	Scaling factor that represents a population’s growth rate as percentage of the mean growth rate of all included populations (0–5 years), estimated using a growth curve model fit for population *i* at time *t*:
	$y_{it}=\alpha_{i}+h[exp(\gamma_{i})\times (t-\beta_{i})]+\varepsilon_{it}$
	Where α_*i*_ is a random height effect for population *i*, *h* is a cubic B-spline function, β_*i*_ is a random age effect, γ_*i*_ is the intensity parameter representing how compressed or expanded the growth rate of one observed population *i* is compared to the global average growth rate, and ε_*it*_ are residuals [14, 15].
	*Conventional interpretation*: The higher the intensity, the higher the population growth rate relative to the global mean growth rate.

The World Health Organization Growth Standards 2006 were used as the reference population for calculating HAZ [3]. Abbreviations: Growth Delay (GD), Height-for-age difference (HAD), Height-for-age z score (HAZ), Super-Imposition by Translation and Rotation (SITAR).

Model-derived metrics were estimated from regression models based on height or HAZ data for children <5y for each survey and were divided into 3 groups: (i) predicted HAZ at specific ages; (ii) regression slopes of HAZ, HAD, or GD as a function of age; and (iii) the SITAR intensity parameter. To generate the type (i) and (ii) model-derived metrics, we fit a linear spline model of HAZ as a function of age for the <5y population in each survey, with a knot at 731 days (2 years of age). We chose to model HAZ as a function of age rather than use raw heights because the HAZ-age slopes were linear and simpler to fit using linear splines than the curvilinear pattern of raw height-age curves [13]. The knot placement at 731 days is consistent with the theory that the critical postnatal period to prevent undernutrition is within the first two years [19]. Predicted mean HAZs at birth, 2 years, and 5 years of age were estimated from the linear regression spline model. HAZ slopes for the 1mo-2y and 2-5y intervals were determined by dividing the difference in predicted HAZ between the beginning and end of each interval by the total duration (in years) of the interval. Predicted HAZs at 1 month, 2 years, and 5 years were each converted to their corresponding HADs (based on the WHO-GS), and then HAD slopes were calculated in a similar manner as HAZ slopes for the same age intervals (1mo-2y and 2-5y). The predicted HAZs from the HAZ-age model were also used to generate height-age estimates at 1 month, 2 years, and 5 years. A population’s observed mean height is located along the median height trajectory of the WHO-GS; the age (of the standard population) at which the mean height is closest to the WHO-GS median is the population’s average ‘height-age’. In the setting of linear growth faltering, the height-age will be younger than the actual chronological age; this discrepancy can be expressed as GD, which is calculated as the arithmetic difference between the chronological age and the estimated height-age [13]. GD slopes were calculated using the same approach as for HAZ and HAD slopes. Since quantifying height-age at <1 month was not feasible using the WHO-GS, GD slopes could not be calculated for a birth-to-2 years interval; therefore, the 1 month to 2-year age interval was used for HAZ, HAD and GD slopes. We used model-predicted HAZ to express mean height at discrete ages; inferences would be the same had we used mean HAD or GD instead of HAZ because these measures are interchangeable when used at single time points [13]. We assumed a 1:1 sex ratio (male:female) for population-level derivation of HAD and GD from the corresponding predicted HAZ. Positive HAZ and HAD slopes represent favorable trajectories (i.e., mean HAZ/HAD are moving up to zero), whereas positive GD slopes are unfavorable (i.e., accrual of a delay). To estimate the SITAR intensity parameter for each DHS survey, we first generated mean heights within 2-month age intervals to create trajectories from birth to 5 years of age. The SITAR growth model was then fit for each DHS survey in comparison to the mean growth curve of all surveys. The intensity parameter, which represents the velocity of a growth curve as a proportion of the velocity of the mean growth curve, was estimated from the SITAR model for each survey as previously described [15]. All descriptive and model-derived metrics were generated accounting for DHS survey sampling design using strata, cluster, and sample weights. HAZ was calculated using the WHO Child Growth Standards STATA igrowup package [20].

### Population indicator selection

Three indicators of population health and development are known to be associated with child growth and nutritional status, and which were available for all survey years, were selected for inclusion in the analysis [8]. Mortality rate under 5 years of age (<5y) was defined as the number of deaths before five years of age per 1000 live births. A country’s economic output was measured by gross domestic product (GDP) per capita adjusted for purchasing power parity in 2017 in constant international dollars. Under 5y mortality and GDP data were obtained from the World Bank [21]. Maternal education was defined as the proportion of women whose highest level of education was secondary school or above; data for maternal education were extracted from each DHS survey.

### Complementary and alternative growth metric selection and validity assessment

Scatterplots with linear fit lines and locally weighted scatterplot smoothing (LOWESS) curves were generated for each metric-metric and metric-indicator comparison. Some relationships demonstrated non-linearity so we used Spearman’s rank correlation coefficients, the absolute values of which (|*r*|) are shown unless otherwise specified (+*r* or–*r*).

Metrics were considered to be potential alternatives to stunting if they were very strongly correlated with stunting prevalence (|*r*| ≥0.95). Those that had weaker correlations (|*r*|<0.95) with stunting were considered to be potentially complementary to stunting. Complementary metrics were considered as having potentially relevant information about linear growth that would not be captured by stunting prevalence alone. We also considered metrics in terms of their suitability as alternatives to stunting prevalence 2-5y, using similar criteria as for stunting prevalence <5y.

Validity assessment was based on the correlation between each population indicator and linear growth metric using the correlations of stunting with the population indicators as benchmarks. Candidate alternative metrics (i.e., |*r*|≥0.95 with stunting) were prioritized if they also performed at least as well or better than stunting in terms of their correlation with the three population indicators. The core assumption underlying these analyses was that anthropometry-derived metrics that provide valid representations of the health of a population should have relatively robust associations with other indicators of population health and development. This assumption is supported by a framework that was developed using evidence from studies that examined cross-national variation in HAZ/stunting via ecological or multi-level analyses [22, 23] and has been published elsewhere [8]. We did not compare correlation coefficients across the different population-level indicators (e.g., correlation of mean HAZ with under 5y mortality versus correlation of mean HAZ with GDP).

Since survey recency may affect the strength of correlation between linear growth metrics and population indicators, we conducted a sensitivity analysis to assess the correlation strength between linear growth metrics and population indicators when including, for each country: 1) only the earliest survey; 2) only the survey closest to 2010, the midpoint of survey year range; or, 3) only the most recent survey. Every country was represented once in each time period. If a country had two surveys that were equidistant from the midpoint (2010), the later survey was included in the midpoint analysis. If a country had only 1 survey, it was included in all analyses.

In secondary analyses, we compared the model-derived interval-specific slope metrics (HAZ, HAD and GD) to their correlations with under-5 stunting, predicted HAZ at the beginning and end of the age intervals, and across the age intervals (1mo-2y versus 2-5y).

SITAR modeling was done in R using the *nlme* package [24] and Tim Cole’s *sitar* package [25]. Other statistical analyses were carried out in Stata version 16.1 MP (College Station, Texas, USA). Some figures were produced using Tableau desktop (2021.1.5) and Microsoft Excel 2019. Code was independently verified by a second data analyst.

## Results

A summary of the surveys included in the analysis is presented in Table 2. There were 156 surveys from 63 countries included (S1 Table). The median (and range) of sample sizes per survey was 5,461 (1,290 to 239,588), and most children were > 2 years of age (Table 2).

**Table 2:** Characteristics of demographic and health surveys included in analyses.

Characteristic	n = 156
Countries, n	63
Surveys per country, n(%)	
1	19 (30%)
2	19 (30%)
>2	25 (40%)
Surveys by calendar period, n(%)	
2000–04	28 (18%)
2005–09	42 (27%)
2010–14	49 (31%)
2015–2020	37 (59%)
Surveys by world region (UNICEF category), n(%)	
East Asia and Pacific	3 (5%)
Eastern Europe and Central Asia	6 (10%)
Latin America and Caribbean	9 (14%)
Middle East and North Africa	3 (5%)
South Asia	5 (8%)
Eastern and Southern Africa	18 (28%)
West and Central Africa	19 (30%)
Children per survey, median (range)	5461 (1290, 239588)
Children by age category as a percent of all children under 5 years of age, median (range)	
< 2 years of age	41 (34, 48)
2–5 years of age	59 (52, 66)
Survey-specific mean height-for-age z-scores, median (range)	-1.33 (-2.24, -0.14)

### Correlations of candidate growth metrics with stunting prevalence <5y

Six of the candidate metrics had correlation coefficients of 0.95 or greater with stunting prevalence <5y and were therefore classified as potential alternatives to stunting (Table 3; S2 Table): 5 descriptive metrics (stunting at 2-5y, mean HAZ <5y and 2-5y, 25^th^ percentile (p25) HAZ <5y and 2-5y) and 1 model-derived metric (predicted HAZ at 2y). These metrics as well as two additional model-derived metrics (predicted HAZ at 5y and GD slope 1m-2y) were highly correlated with stunting 2-5y with |*r*| ≥ 0.95 (Table 3; S2 Table). As with predicted HAZ at 5y and GD slope 1m-2y, the remaining 7 metrics had |r| < 0.95 with stunting prevalence and were therefore considered as potential complementary metrics (Table 3; S2 Table). Among these, SITAR-IP had the strongest correlation with stunting prevalence <5y (|*r*| = 0.88) while HAZ slope 2-5y and predicted HAZ at birth had the weakest correlations ((|*r*| = 0.05 and 0.32, respectively). In a post-hoc analysis including each country’s most recent survey, country rankings differed based on the metric, particularly among metrics considered potentially complementary (S2 Fig).

**Table 3:** Correlations between candidate linear growth metrics.

		<5 years					2–5 years							1 month– 2 years			
		Stunting Prev.	Mean HAZ	p25 HAZ	SITAR IP	Pred. HAZ 0y	Stunting Prev.	Mean HAZ	p25 HAZ	HAZ slope	HAD slope	GD slope	Pred. HAZ 5y	HAZ slope	HAD slope	GD slope	Pred. HAZ 2y
**<5 years**	Stunting Prev.	-	-0.97	-0.99	-0.88	-0.32	0.99	-0.97	-0.97	0.05^ns^	-0.62	0.86	-0.94	-0.69	-0.83	0.94	-0.95
	Mean HAZ	-0.97	-	0.94	0.86	0.42	-0.96	0.98	0.93	-0.08^ns^	0.60	-0.86	0.95	0.64	0.80	-0.94	0.96
	p25 HAZ	-0.99	0.94	-	0.88	0.27	-0.98	0.95	0.99	-0.07^ns^	0.59	-0.84	0.93	0.71	0.84	-0.93	0.93
	SITAR IP	-0.88	0.86	0.88	-	0.02^ns^	-0.93	0.93	0.92	0.13^ns^	0.73	-0.92	0.95	0.81	0.87	-0.88	0.86
	Pred. HAZ 0y	-0.32	0.42	0.27	0.02^ns^	-	-0.24	0.26	0.18	<0.01^ns^	0.19	-0.25	0.26	-0.35	-0.12^ns^	-0.16	0.25
**2–5 years**	Stunting Prev.	0.99	-0.96	-0.98	-0.93	-0.24	-	-0.99	-0.98	0.03^ns^	-0.65	0.89	-0.97	-0.74	-0.87	0.95	-0.95
	Mean HAZ	-0.97	0.98	0.95	0.93	0.26	-0.99	-	0.96	-0.04^ns^	0.64	-0.89	0.97	0.74	0.87	-0.96	0.97
	p25 HAZ	-0.97	0.93	0.99	0.92	0.18	-0.98	0.96	-	-0.04^ns^	0.62	-0.87	0.95	0.76	0.87	-0.94	0.93
	HAZ slope	0.05^ns^	-0.08^ns^	-0.07^ns^	0.13^ns^	<0.01^ns^	0.03^ns^	-0.04^ns^	-0.04^ns^	-	0.71	-0.37	0.15^ns^	-0.25	-0.27	0.27	-0.26
	HAD slope	-0.62	0.60	0.59	0.73	0.19	-0.65	0.64	0.62	0.71	-	-0.91	0.79	0.31	0.38	-0.44	0.45
	GD slope	0.86	-0.86	-0.84	-0.92	-0.25	0.89	-0.89	-0.87	-0.37	-0.91	-	-0.97	-0.57	-0.67	0.75	-0.76
	Pred. HAZ 5y	-0.94	0.95	0.93	0.95	0.26	-0.97	0.97	0.95	0.15^ns^	0.79	-0.97	-	0.68	0.80	-0.89	0.89
**1 month—2 years**	HAZ slope	-0.69	0.64	0.71	0.81	-0.35	-0.74	0.74	0.76	-0.25	0.31	-0.57	0.68	-	0.96	-0.83	0.77
	HAD slope	-0.83	0.80	0.84	0.87	-0.12^ns^	-0.87	0.87	0.87	-0.27	0.38	-0.67	0.80	0.96	-	-0.94	0.91
	GD slope	0.94	-0.94	-0.93	-0.88	-0.16	0.95	-0.96	-0.94	0.27	-0.44	0.75	-0.89	-0.83	-0.94	-	-0.99
	Pred. HAZ 2y	-0.95	0.96	0.93	0.86	0.25	-0.95	0.97	0.93	-0.26	0.45	-0.76	0.89	0.77	0.91	-0.99	-
		**<5 years**					**2–5 years**							**1 month– 2 years **			

N = 156 surveys. Grey shaded cells have an absolute Spearman’s correlation coefficient ≥ 0.95. Abbreviations: 25th percentile (p25), Growth Delay (GD), Height-for-age difference (HAD), Height-for-age z score (HAZ), Month (m), Predicted (Pred.), Prevalence (Prev.), Super-Imposition by Translation And Rotation Intensity Parameter (SITAR-IP), Year (y)

^ns^ Non-significant Spearman’s correlation coefficient p ≥ 0.05. All other coefficients are significant with p < 0.05.

### Correlations of linear growth metrics with indicators of population health and development

**Fig 2:**
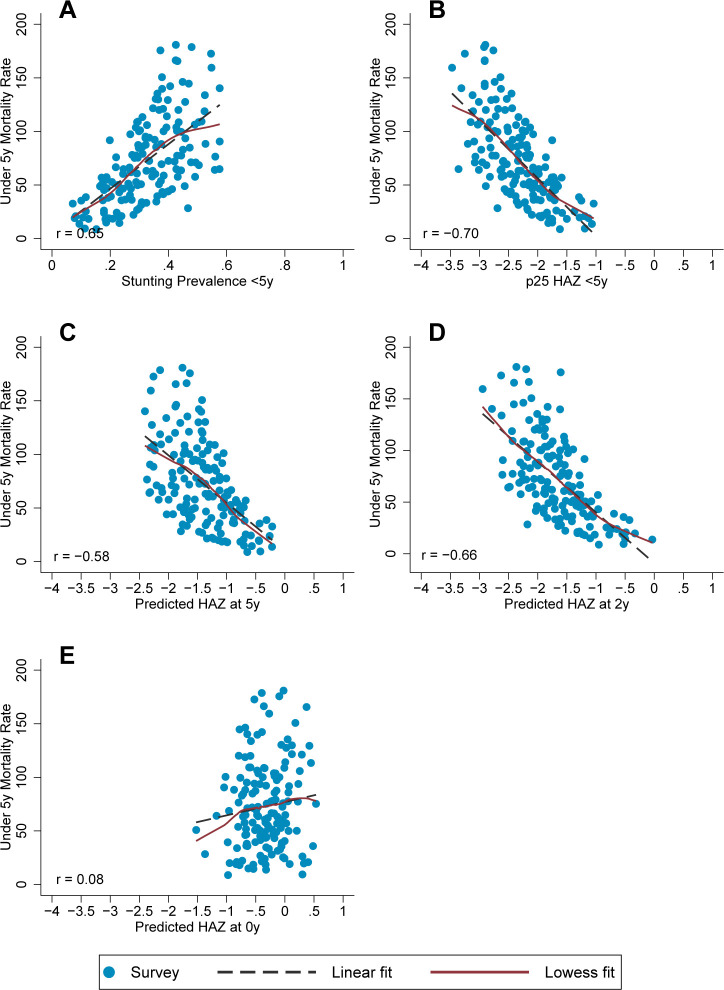
Relationships between under 5y mortality rate and linear growth metrics. Selected metric-indicator relationships show varying strengths of correlation of linear growth metrics with under 5y mortality rate (defined as the number of deaths before five years of age per 1000 live births). Each blue circle represents one Demographic and Health Survey (N = 156). Abbreviations: Height-for-age difference (HAD), Height-for-age z score (HAZ), Month (m), year (y).

**Fig 3:**
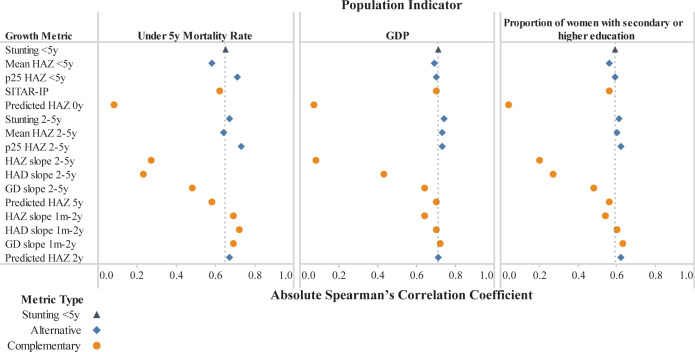
Correlations of candidate linear growth metrics with population indicators (N = 156 surveys). Under 5y mortality rate defined as the number of deaths before five years of age per 1000 live births. GDP defined per capita adjusted for purchasing power parity in 2017 in constant international dollars. A vertical dotted line represents the correlation of each population indicator with stunting among those under 5 years of age. Abbreviations: 25th percentile (p25), Growth Delay (GD), Height-for-age difference (HAD), Height-for-age z score (HAZ), Month (m), Super-Imposition by Translation and Rotation Intensity Parameter (SITAR-IP), year (y).

Correlations between stunting prevalence <5y and the population indicators were +0.65 for under 5y mortality, –0.71 for GDP, and –0.59 for maternal education (Figs 2 and 3; S3 Table**)**. Compared to stunting, all six candidate alternative metrics had similar and, in several instances, slightly stronger correlations with the population indicators (Fig 3). For 6 of the 9 candidate complementary metrics (SITAR-IP, predicted HAZ at 5y, HAZ slope 1 m-2y, HAD slope 1m-2y, GD slopes 1m-2y and 2-5y), the magnitudes of their correlations with the population indicators ranged from 0.48 to 0.72 for under 5y mortality, 0.64 to 0.72 for GDP, and 0.48 to 0.63 for maternal education (Fig 3; S3 Table). Two of those 6 complementary metrics (predicted HAZ at 2y, GD slope 1m-2y) had correlations that were generally stronger than those of stunting prevalence <5y, and SITAR-IP was all similar to stunting prevalence <5y (Fig 3; S3 Table). The other 3 of 9 candidate complementary metrics (HAZ slope 2-5y, HAD slope 2-5y, and predicted HAZ at birth) had consistently lower magnitudes of correlation with the population indicators; in particular, predicted HAZ at birth was an outlier with correlation magnitudes all less than 0.1 (Figs 2 and 3 and S3 Table).

Sensitivity analyses demonstrated a pattern of mostly weakening correlations of linear growth metrics with under 5y mortality and GDP from earliest to most recent surveys, whereas many of the relationships with maternal education were more stable over time (S3 Fig). Predicted HAZ at birth and HAZ slope 2-5y were exceptions, as several of their correlations with under 5y mortality and GDP were of higher magnitude in the most recent surveys compared to earlier surveys (S3 Fig).

### Comparisons among age interval-specific HAZ, HAD and GD slopes

In further comparisons of the model-derived slopes, GD slopes in both age intervals had slightly stronger correlations with stunting prevalence <5y (+0.94 and +0.86 for 1m-2y and 2-5y, respectively) than HAD slopes (–0.83 and –0.62 for 1m-2y and 2-5y, respectively) (Table 3; S1 Fig). HAZ slope 1m-2y had a robust association with stunting prevalence <5y in the expected direction (*r* = –0.69) but HAZ slope 2-5y was not correlated with stunting prevalence <5y (*r* = +0.05) (Table 3; S1 Fig**)**. Correlations of HAZ slope 2-5y with the population indicators were weaker than those for HAD and GD slopes 2-5y, and had opposite interpretations (S3 Table**)**.

Correlations of the slope metrics with predicted HAZ at the beginning and end of the corresponding age intervals varied in magnitude and direction (Table 3). For the 1m-2y age interval, the slope metrics had weaker correlations with predicted HAZ at birth (0.12 ≤ |*r*| ≤ 0.35) than predicted HAZ at 2y (0.77 ≤ |*r*| ≤ 0.99). The direction of these correlations changed from negative at birth to positive at 2y for both HAZ and HAD slopes, whereas correlations with GD slopes were negative for both time points (i.e., progressive faltering defined as an increase in GD was associated with lower mean HAZ at birth and 2 years) (Table 3). HAZ slope 2-5y had a weak correlation that was inverse (*r* = -0.26) for predicted HAZ at 2y and positive (+0.15) for predicted HAZ at 5y. Correlations between HAD slope 2-5y and predicted HAZ at 2y and 5y were stronger and positive (+0.45 and +0.79, respectively), and GD slope 2-5y had stronger negative correlations with predicted HAZ at 2y and 5y (–0.76 and –0.97, respectively) (Table 3). When comparing each slope metric between the two age intervals, HAZ slope 1m-2y was inversely correlated with HAZ slope 2-5y, whereas the two slopes were positively correlated for both HAD and GD, though more strongly for GD (Table 3; S1 Fig). SITAR-IP was uncorrelated with predicted HAZ at birth but had strong correlations with both predicted HAZ at 2y (+0.86) and 5y (+0.95), and directions consistent with HAD and GD slopes in both intervals (Table 3).

## Discussion

The prevalence of under-5 stunting is a widely accepted indicator of the nutritional and health status of children in LMICs and is currently used to determine whether countries are on- or off-track with respect to achieving the WHO 2025 nutrition targets and SDGs [1, 4]. However, stunting is based on a statistical rather than biological cut-point in the population-level height-for-age distribution and is therefore prone to misinterpretation as a clinical classifier, rather than its intended use as a statistical measure of location of an observed height-age distribution compared to a healthy standard. In this study based on aggregated DHS datasets from 63 LMICs, we examined 15 other candidate linear growth metrics that can be derived from anthropometric survey data and the same height-for-age distributions from which stunting is estimated. Building on prior work [8], we considered metrics highly correlated with stunting (|*r*| ≥0.95) as alternatives to stunting whereas those with somewhat weaker correlations may serve in a complementary role. We applied a straightforward framework to validate the metrics by considering their correlations with three established indicators of population health and development: under 5y mortality, GDP, and maternal education.

Stunting prevalence <5y was relatively strongly correlated with population indicators, affirming its validity as an indicator of the health and well-being of children in LMICs. The findings did not support our hypothesis that stunting would be unduly influenced by errors in the height or age data compared to measures of central tendency. However, several of the other metrics performed as well or better than stunting in this study framework. Six of 15 metrics met our criteria as alternatives to stunting prevalence, and 6 others were promising candidates as complements. The marked variability in country rankings among the complementary metrics highlighted the extent to which they reflected different dimensions of the same underlying problem. In particular, model-derived metrics such as GD slopes and SITAR-IP may demonstrate between-survey variations in the age-related dynamics of child growth that are not revealed by stunting prevalence <5y or other descriptive statistics. The present findings suggest these metrics may be considered for use alongside stunting to track progress in advancing child growth and nutrition.

Several of our findings were unsurprising because standardized height distributions are nearly symmetrical and increases in the proportion of HAZ < -2 generally reflect the negative shift of the whole height distribution rather than a left skewing [6, 7, 26]. As previously established [8], stunting prevalence <5y and mean HAZ <5y were highly correlated and had similar magnitudes of correlations with population indicators. Briend et al. compared stunting to mean HAZ in a cohort in Bangladesh and found the two indicators similarly reflected seasonal and secular changes in nutritional status, although detecting differences in mean HAZ required smaller survey sample sizes than stunting [27]. In its 1995 technical report, the WHO Expert Committee on Physical Status elaborated on the conceptual and statistical advantages of using mean HAZ instead of stunting prevalence to describe populations [7]. However, we are not aware of prior studies that have empirically examined a range of statistics to summarize length/height distributions or considered their ecological associations with health or socioeconomic indicators.

The p25 HAZ metric was found to track closely with stunting prevalence <5y and mean HAZ <5y; its correlation may have been stronger with stunting prevalence <5y than with mean HAZ <5y because both p25 and stunting reflect the left tail of the distribution and do not rely on an assumption of normality. Stunting prevalence, mean HAZ and p25 HAZ limited to the 2-5y age range were all highly correlated with stunting prevalence <5y, perhaps partly because most surveyed children under 5 years of age were >2 years of age. p25 HAZ <5y performed at least as well as stunting prevalence <5y in the validation exercise while p25 HAZ 2-5y generally outperformed both stunting prevalence <5y and 2-5y. Like mean HAZ, p25 HAZ reflects the downward shift of the entire HAZ distribution and thereby conveys the notion of linear growth faltering as a pervasive community problem affecting nearly all children in LMICs. However, communicating the p25 HAZ metric may be challenging (e.g., its benchmark value for a healthy reference population is −0.675), and mean HAZ may be more appealing as it is nearly as robust as p25 HAZ and easier to interpret because it is a measure of central tendency, whereby any value below 0 indicates that the distribution is left-shifted and therefore the population has experienced linear growth faltering [7].

Longitudinal birth cohort studies have shown associations of birth length with neonatal and infant mortality [28] and a substantial fraction of postnatal linear growth faltering is already present at birth [29]. However, in the present study, predicted HAZ at birth performed poorly both in terms of its correlation with stunting prevalence <5y and its relationship to population indicators. Greater variance in early infant length measurements has been observed in DHS datasets [6, 30, 31], suggesting that weak ecological associations of early infant length-based metrics with population indicators may be due to greater anthropometric measurement error at young ages. The poor performance of predicted HAZ at birth may also be due to the selection of population indicators in this study; HAZ at birth may perform better if assessed in relation to other indicators such as neonatal mortality, neurodevelopmental, or school-related outcomes [32].

Predicted HAZ at 2y and 5y both performed well in the present framework and could serve as alternatives to stunting as they share the same advantage with other measures of central tendency in correctly reflecting a whole-population shift. Although we expressed these age-specific means on the HAZ scale, our prior work demonstrated that inferences would be unchanged if these estimates were re-scaled as HAD or GD, since the three measures are nearly perfectly correlated when calculated at discrete ages [13]. We previously reasoned that GD is conceptually preferred because it reflects the underlying biological process of delayed skeletal maturation and yields coherent statements about the severity of growth faltering based on delays on the age scale rather than deficits in stature or z-scores [13], the latter of which are unlikely to be intuitive for most lay audiences. Consideration may therefore be given to the adoption of population-average GD at 2 and/or 5 years as alternatives to stunting prevalence <5y for tracking between- and within-population differences in linear growth. Predicted GD at 5 years of age may be a particularly relevant metric where policies or programs target the growth of preschool-aged children beyond the first thousand days of life.

Our previous work compared patterns of change in GD with age with those of HAZ and HAD and presented a theoretical rationale for selecting GD over HAZ or HAD to describe population-averaged linear growth trajectories [13]. Whereas HAZ and HAD are based on comparisons to a reference population of the same chronological age, GD is a comparison to healthy children of the same height-age; as growth faltering proceeds, chronological age diverges from biological age (i.e. progressively lower height-ages compared to the chronological age-matched reference population, yielding greater GD). The present study provides new empirical support for the preference for GD to represent linear growth trajectories. For the 1mo-2y age period, when faltering is typically most pronounced regardless of which slope metric is used [13], all 3 slope metrics were similarly correlated with the population indicators (with magnitudes similar to those observed for stunting prevalence <5y). However, in the 2-5y age period, GD slope outperformed the other slope metrics in the present validation framework. HAZ slope 2-5y had weak correlations with the population indicators and the directions were counter-intuitive (i.e., an improving mean HAZ–increasing towards zero–was correlated with higher mortality, lower GDP and a lower proportion of women with secondary/higher education). Furthermore, in sensitivity analyses, we found the correlations of HAZ slope 2-5y with under 5y mortality and GDP were stronger in more recent surveys, contrary to our expectation that these correlations would attenuate as health and economic status improves (which is what was observed for stunting and many other metrics). Overall, these findings are consistent with previous work showing that the use of mean HAZ to portray population-average faltering (HAZ-tracking) may be problematic because the linear growth of children in a population can be sub-normal yet appear faster than that of healthy children of the same starting height and same chronological age [13]. HAZ-tracking classically suggests a plateau or recovery of growth beyond 2 years of age in many LMICs where a mean HAZ slope greater than 1 (i.e., upward, towards zero) is interpreted as catch-up growth [33]. However, both HAD and GD clearly show continued faltering beyond 2 years of age [13], an inference reinforced by the strong correlations of HAD and GD slopes between the two adjacent age intervals (1m-2y versus 2-5y) and the strong correlations of HAD and GD slopes with SITAR-IP and predicted HAZ at the end of each interval. In contrast, use of HAZ slopes indicated that more severe faltering at <2y correlates with faster catch-up from 2-5y (possibly a manifestation of regression to the mean) [33] and, counter-intuitively, that faster catch-up from 2-5y is unrelated to either predicted HAZ 5y or stunting prevalence 2-5y. The conceptual pitfalls of HAZ-tracking combined with the present empirical results put into question the widespread use of HAZ-by-age graphs (sometimes referred to as ‘Victora plots’ [34]) to make within- and between-country comparisons of the age-related dynamics of linear growth faltering.

Frameworks to judge the validity of novel health measures have generally focused on individual-level classification (e.g., screening, intervention response) applicable to clinical settings or epidemiological studies rather than population-average summaries of cross-sectional survey data. Here, the validity assessment relied on ecological-level correlations with specific indicators of population well-being to which child nutritional status is expected to be related. We acknowledge this approach had a narrow scope and that use of other indicators of health, socioeconomic status, or within-country inequalities may have yielded different conclusions. However, the availability of population-representative measures of some of the other relevant domains (e.g., cognitive development, school readiness) was limited in DHS surveys or not available from other external data sources for all countries and survey years included in our analyses [8]. Future work should consider other statistical properties of the examined linear growth metrics; for example, variance (standard error) will influence the precision with which a metric enables between-population comparisons or tracking secular changes. Another key consideration for future studies is the acceptability and/or interpretability of metrics for stakeholders. Among advocates and policymakers, the familiar use of stunting has brought attention to the problem of childhood undernutrition in LMICs [4], even though the prevalence of stunting under-estimates the total burden of undernutrition [26]. Furthermore, extensive work has already established methods for measuring secular trends in stunting prevalence [4] and set thresholds to prompt policy action [5]. Adoption of alternative and complementary metrics would require similar efforts to define benchmarks and establish guidance for their interpretation as health and nutrition indicators. Even if there is openness to new linear growth metrics, a recent USAID report cited concerns about the low responsiveness of height-based measures to interventions (i.e., failure to reduce stunting does not equate to a lack of benefit for other outcomes) and recommended a broader set of indicators such as diet quality, well-being, and health status [35]. Therefore, future research would ideally examine the responsiveness of the proposed metrics to interventions and the extent to which their use might differentially influence decision-making in diverse programming and policy-making contexts [7, 36], including humanitarian emergencies and timely warning systems.

There are other limitations of this study that should be acknowledged. First, the performance of the model-derived metrics may have been influenced by the regression modeling approach. We generated HAZ, HAD and GD metrics from the same underlying HAZ-age functions to promote their direct comparability, and included all data from 0 to 5 years in one model with spline terms. In future applications, consideration may be given to modeling HAD and GD separately or using distinct models for each age interval. Second, the number of surveys was too low to precisely examine differences across world regions or other country-level characteristics. Third, we did not examine variations in survey quality as a modifier of metric-indicator correlations. In prior work, we explored whether an index of anthropometric survey quality affected metric performance [37]; however, the approach was not included in the current study, primarily because survey quality is itself associated with the burden of growth faltering.

In conclusion, we identified several linear growth metrics derived from anthropometric survey data that could be adopted for tracking global progress in child health and nutritional status. Stunting, which was found to be a valid method of summarizing population height distributions, has the distinct advantages of being straightforward to estimate and familiar to policymakers. However, alternative metrics such as mean HAZ or p25 HAZ may offer conceptual advantages, and others may provide complementary information about population-average age-related dynamics of linear growth (e.g., GD slope 2-5y, SITAR-IP). The potential utility of these alternative and complementary linear growth metrics warrants further research and validation in real-world applications, including assessments of their acceptability and ease of interpretation by stakeholders.

## Supporting information

S1 Fig Scatterplots of relationships between linear growth metrics. Selected metric-metric relationships shown here demonstrate the relationships of slope metrics between the 1m-2y and 2-5y age ranges (both shown in units of HAZ) and the relationships of the slopes in the 2-5y range with stunting prevalence <5y (shown as a proportion). Each blue circle represents one Demographic and Health Survey (N = 156. Abbreviations: Growth Delay (GD), Height-for-age difference (HAD), Height-for-age z score (HAZ), Month (m), year (y).
(PDF)

S2 Fig Ranking of countries by metric for the 10 countries with the lowest and 10 countries with the highest stunting prevalence. Panel A, 10 countries with the lowest stunting prevalence. Panel B, 10 countries with the highest stunting prevalence. The most recent survey for each country was used (N = 63). Surveys were ranked based on the estimated metric value. Larger values of <5y mean HAZ, <5y p25 HAZ, 2-5y mean HAZ, 2-5y p25 HAZ, predicted HAZ at 2y, <5y SITAR-IP, predicted HAZ at 5y, 1m-2y HAD slope, predicted HAZ at birth, 2-5y HAZ slope, and 2-5y HAD slope indicate less growth faltering and are ranked closer to 1. Larger values of <5y stunting, 2-5y stunting, 2-5y GD slope, and 1m-2y GD slope indicate greater growth faltering and are ranked further from 1. Candidate alternative metrics have a ≥|0.95| Spearman correlation with stunting <5y and an absolute Spearman correlation with under 5y mortality, gross domestic product, and the proportion of women with secondary education or higher that is the same or higher than the correlation of stunting <5y with these 3 population health indicators. Candidate complementary metrics have a ≥|0.95| Spearman correlation with stunting <5y and are moderately correlated with the 3 population health indicators. Other metrics are those which did not meet the criteria for candidate alternative or complementary. Abbreviations: Growth Delay (GD), Height-for-age difference (HAD), Height-for-age z score (HAZ), Month (m), year (y).
(DOCX)

S3 Fig Time trends of correlations of candidate linear growth metrics with population indicators. Each analysis included one selected Demographic and Health survey from each country (N = 63) that was either the ‘earliest or only’ survey, the survey closest to the midpoint year (2010), or the ‘more recent or only’ survey. Under 5y mortality rate defined as the number of deaths before five years of age per 1000 live births. GDP defined per capita adjusted for purchasing power parity in 2017 in constant international dollars. Abbreviations: Growth Delay (GD), Height-for-age difference (HAD), Height-for-age z score (HAZ), Month (m), year (y).
(DOCX)

S1 Table Demographic and health surveys included in analyses. (PDF)

S2 Table Pairwise correlations between linear growth metrics. Values shown are Spearman’s correlation coefficient (95% confidence interval), n = 156 surveys. These are the same data shown in Table 3 but the 95% confidence intervals are additionally shown here. Grey shaded cells have an absolute correlation coefficient of ≥ 0.95. Abbreviations: 25^th^ percentile (p25), Growth delay (GD), Height-for-age difference (HAD), Height-for-age z score (HAZ), Month (m), Predicted (Pred), Prevalence (Prev.), Super Imposition by Translation and Rotation Intensity Parameter (SITAR-IP), year (y).
(PDF)

S3 Table Correlations between linear growth metrics and population indicators (N = 156 demographic and health surveys). Values shown are Spearman’s correlation coefficient (95% confidence interval). Grey shaded cells are considered as candidate alternative metrics to stunting prevalence (absolute Spearman correlation coefficient with under 5y stunting prevalence is ≥ 0.95). Under 5y mortality rate defined as the number of deaths before five years of age per 1000 live births. GDP defined per capita adjusted for purchasing power parity in 2017 in constant international dollars. Abbreviations: 25^th^ percentile (p25), Growth delay (GD), Height-for-age difference (HAD), Height-for-age z score (HAZ), Month (m), Predicted (Pred), Prevalence (Prev.), Super Imposition by Translation and Rotation Intensity Parameter (SITAR-IP), year (y).
(PDF)

S1 File Instructions for accessing the specific datasets and variables used in this study. (DOCX)

## References

[1] World Health Organization. Health in 2015: from MDGs, Millennium Development Goals to SDGs Sustainable Development Goals [Internet]. 2015 [cited S2022 Sep 19]. Available from: https://apps.who.int/iris/handle/10665/200009.

[2] Victora CG, Christian P, Vidaletti LP, Gatica-Domínguez G, Menon P, Black RE. Revisiting maternal and child undernutrition in low-income and middle-income countries: variable progress towards an unfinished agenda. The Lancet. 2021;397:1388–99. doi: 10.1016/S0140-6736(21)00394-9 33691094 PMC7613170

[3] W. H. O. Multicentre Growth Reference Study Group. WHO Child Growth Standards based on length/height, weight and age. Acta Paediatr Suppl. 2006;450:76–85. doi: 10.1111/j.1651-2227.2006.tb02378.x 16817681

[4] de Onis M, Dewey KG, Borghi E, Onyango AW, Blössner M, Daelmans B, et al. The world health organization’s global target for reducing childhood stunting by 2025: Rationale and proposed actions. Maternal and Child Nutrition. 2013;9(S2):6–26. doi: 10.1111/mcn.12075 24074315 PMC6860845

[5] de Onis M, Borghi E, Arimond M, Webb P, Croft T, Saha K, et al. Prevalence thresholds for wasting, overweight and stunting in children under 5 years. Public Health Nutrition. 2019;22(1):175–9. doi: 10.1017/S1368980018002434 30296964 PMC6390397

[6] Roth DE, Krishna A, Leung M, Shi J, Bassani DG, Barros AJD. Early childhood linear growth faltering in low-income and middle-income countries as a whole-population condition: analysis of 179 Demographic and Health Surveys from 64 countries (1993–2015). The Lancet Global Health. 2017;5(12):e1249–e57. doi: 10.1016/S2214-109X(17)30418-7 29132614 PMC5695758

[7] WHO Expert Committee on Physical Status. Physical Status, the use and interpretation of anthropometry: report of a WHO expert committee [Internet]. 1995 [cited 2022 Sep 19]. Available from: https://apps.who.int/iris/handle/10665/37003.

[8] Aimone A, Bassani DG, Qamar H, Perumal N, Namaste S, Roth DE. Alternative and Complementary Metrics of Linear Growth for Tracking Global Progress in Child Nutritional Status. 2021. DHS Working Paper No. 153. Rockville, Maryland, USA: ICF.

[9] The DHS Program. Biomarker Field Manual [Internet]. 2021 [cited 2022 Sep 19]. Available from: https://www.dhsprogram.com/publications/publication-dhsm7-dhs-questionnaires-and-manuals.cfm.

[10] Corsi DJ, Neuman M, Finlay JE, Subramanian SV. Demographic and health surveys: A profile. International Journal of Epidemiology. 2012;41(6):1602–13. doi: 10.1093/ije/dys184 23148108

[11] The DHS Program. Guide to DHS Statistics (English) [Internet]. 2018 [cited 2022 Sep 27]. Available from: https://dhsprogram.com/publications/publication-dhsg1-dhs-questionnaires-and-manuals.cfm.

[12] Leroy JL, Ruel M, Habicht J-P, Frongillo EA. Using height-for-age differences (HAD) instead of height-for-age z-scores (HAZ) for the meaningful measurement of population-level catch-up in linear growth in children less than 5 years of age. BMC pediatrics. 2015;15(1):1–11. doi: 10.1186/s12887-015-0458-9 26444012 PMC4595313

[13] Mansukoski L, Qamar H, Perumal N, Aimone A, Bassani DG, Roth DE. Growth delay: an alternative measure of population health based on child height distributions. Annals of Human Biology. 2022;49(2):100–8. doi: 10.1080/03014460.2022.2091794 35736806

[14] Cole TJ, Donaldson MDC, Ben-shlomo Y. SITAR-a useful instrument for growth curve analysis. International Journal of Epidemiology. 2010;39(6):1558–66. doi: 10.1093/ije/dyq115 20647267 PMC2992626

[15] Ohuma EO, Bassani DG, Qamar H, Yang S, Roth DE. A novel development indicator based on population-average height trajectories of children aged 0–5 years modelled using 145 surveys in 64 countries, 2000–2018. BMJ Global Health. 2021;6. doi: 10.1136/bmjgh-2020-004107 33648981 PMC7925247

[16] Gausman J, Kim R, Li Z, Tu L, Rajpal S, Joe W, et al. Comparison of Child Undernutrition Anthropometric Indicators Across 56 Low- and Middle-Income Countries. JAMA Network Open. 2022;5(3). doi: 10.1001/jamanetworkopen.2022.1223 35275168 PMC8917428

[17] Frongillo EA, Baranowski T, Subar AF, Tooze JA, Kirkpatrick SI. Establishing Validity and Cross-Context Equivalence of Measures and Indicators. Journal of the Academy of Nutrition and Dietetics. 2019;119(11):1817–30. doi: 10.1016/j.jand.2018.09.005 30470590

[18] World Health Organization. Computation of centiles and z-scores for height-for-age, weight-for-age and BMI-for-age [Internet]. 2007 [cited 2022 Sep 19]. Available from: https://cdn.who.int/media/docs/default-source/child-growth/growth-reference-5-19-years/computation.pdf?sfvrsn=c2ff6a95_4.

[19] Black RE, Victora CG, Walker SP, Bhutta ZA, Christian P, De Onis M, et al. Maternal and child undernutrition and overweight in low-income and middle-income countries. The Lancet. 2013;382:427–51. doi: 10.1016/S0140-6736(13)60937-X 23746772

[20] World Health Organization. WHO Child Growth Standards: STATA igrowup package.[software]. 2021 [cited 2021 Sep 10]. Available from: https://www.who.int/tools/child-growth-standards/software.

[21] World Bank. World Bank Open Data Indicators [Internet]. 2021 [cited 2021 Nov 16] Available from: https://data.worldbank.org/indicator.

[22] Frongillo EA, de Onis M, Hanson KMP. Socioeconomic and demographic factors are associated with worldwide patterns of stunting and wasting of children. The Journal of Nutrition. 1997;127(12):2302–9. doi: 10.1093/jn/127.12.2302 9405578

[23] Milman A, Frongillo EA, de Onis M, Hwang JY. Differential improvement among countries in child stunting is associated with long-term development and specific interventions. The Journal of Nutrition. 2005;135(6):1415–22. doi: 10.1093/jn/135.6.1415 15930446

[24] Pinheiro J, Bates D, R Core Team. nlme: linear and nonlinear mixed effects models. Version 3.1–162 [software]. 2019 [cited 2021 Aug 12]. Available from:https://cran.r-project.org/package=nlme.

[25] Cole T. Super Imposition by Translation and Rotation Growth Curve Analysis. Version 1.3.0 [software]. 2021 [cited 2021 Aug 12]. Available from: https://rdrr.io/cran/sitar/.

[26] Perumal N, Bassani DG, Roth DE. Use and misuse of stunting as a measure of child health. Journal of Nutrition. 2018;148(3):311–5. doi: 10.1093/jn/nxx064 29546307

[27] Briend A, Henry F. Measuring Change in Nutritional Status: A Comparison of Different Anthropometric Indices and the Sample Sizes Required. European Journal of Clinical Nutrition. 1989;43:769–78. 2627925

[28] Kang Y, Wu LSF, Shaikh S, Ali H, Shamim AA, Christian P, et al. Birth anthropometry predicts neonatal and infant mortality in rural Bangladesh: a focus on circumferential measurements. American Journal of Clinical Nutrition. 2022;115(5):1334–43. doi: 10.1093/ajcn/nqab432 35021206 PMC9071409

[29] Christian P, Lee SE, Angel MD, Adair LS, Arifeen SE, Ashorn P, et al. Risk of childhood undernutrition related to small-for-gestational age and preterm birth in low- and middle-income countries. International Journal of Epidemiology. 2013;42(5):1340–55. doi: 10.1093/ije/dyt109 23920141 PMC3816349

[30] World Health Organization and the United Nations Children’s Fund (UNICEF). Recommendations for data collection, analysis and reporting on anthropometric indicators in children under 5 years old [Internet]. 2019 [cited 2022 Sep 19]. Available from: https://www.who.int/publications/i/item/9789241515559.

[31] Assaf S, Kothari M, Pullum T. An assessment of the quality of DHS anthropometric data, 2005–2014. 2015. DHS Methodological Reports No 16. Rockville (Maryland): ICF International.

[32] Poveda NE, Hartwig FP, Victora CG, Adair LS, Barros FC, Bhargava SK, et al. Patterns of Growth in Childhood in Relation to Adult Schooling Attainment and Intelligence Quotient in 6 Birth Cohorts in Low- A nd Middle-Income Countries: Evidence from the Consortium of Health-Oriented Research in Transitioning Societies (COHORTS). Journal of Nutrition. 2021;151(8):2342–52. doi: 10.1093/jn/nxab096 33982126 PMC8436131

[33] Cameron N, Preece MA, Cole TJ. Catch-up growth or regression to the mean? Recovery from stunting revisited. American Journal of Human Biology. 2005;17(4):412–7. doi: 10.1002/ajhb.20408 15981181

[34] Victora CG, de Onis M, Hallal PC, Blössner M, Shrimpton R. Worldwide timing of growth faltering: revisiting implications for interventions. Pediatrics. 2010;125(3):e473–e80-e–e80. doi: 10.1542/peds.2009-1519 20156903

[35] USAID Advancing Nutrition. Stunting: Considerations for Use as an Indicator in Nutrition Projects [Internet]. 2020 [cited 2022 Sep 19]. Available from: https://www.advancingnutrition.org/resources/stunting-considerations-use-indicator-nutrition-projects.

[36] Habicht J, Pelletier DL, Guthrie HA, Martin RJ, Meyers LD, Olson JA, et al. The Importance of Context in Choosing Nutritional Indicators. The Journal of Nutrition. 1990;120:1519–24. doi: 10.1093/jn/120.suppl_11.1519 2243298

[37] Perumal N, Namaste S, Qamar H, Aimone A, Bassani DG, Roth DE. Anthropometric data quality assessment in multisurvey studies of child growth. The American journal of clinical nutrition. 2020;112(Supplement_2):806S–15S-S–15S. doi: 10.1093/ajcn/nqaa162 32672330 PMC7487428

