# Supplementary material for: Complementary and alternative metrics for tracking population-level trends in child linear growth
Source: PLOS Glob Public Health. 2023 Apr 17;3(4):e0001766. doi: 10.1371/journal.pgph.0001766 (PMC10109512; doi:10.1371/journal.pgph.0001766)

S1 Fig. Scatterplots of relationships between linear growth metrics. Selected metric-metric relationships shown here demonstrate the relationships of slope metrics between the 1m-2y and 2-5y age ranges (both shown in units of HAZ) and the relationships of the slopes in the 2-5y range with stunting prevalence among children under 5 years of age (shown as a proportion). Each blue circle represents one Demographic and Health Survey (N = 156. Abbreviations: Growth Delay (GD), Height-for-age difference (HAD), Height-for-age z score (HAZ), Month (m), year (y)

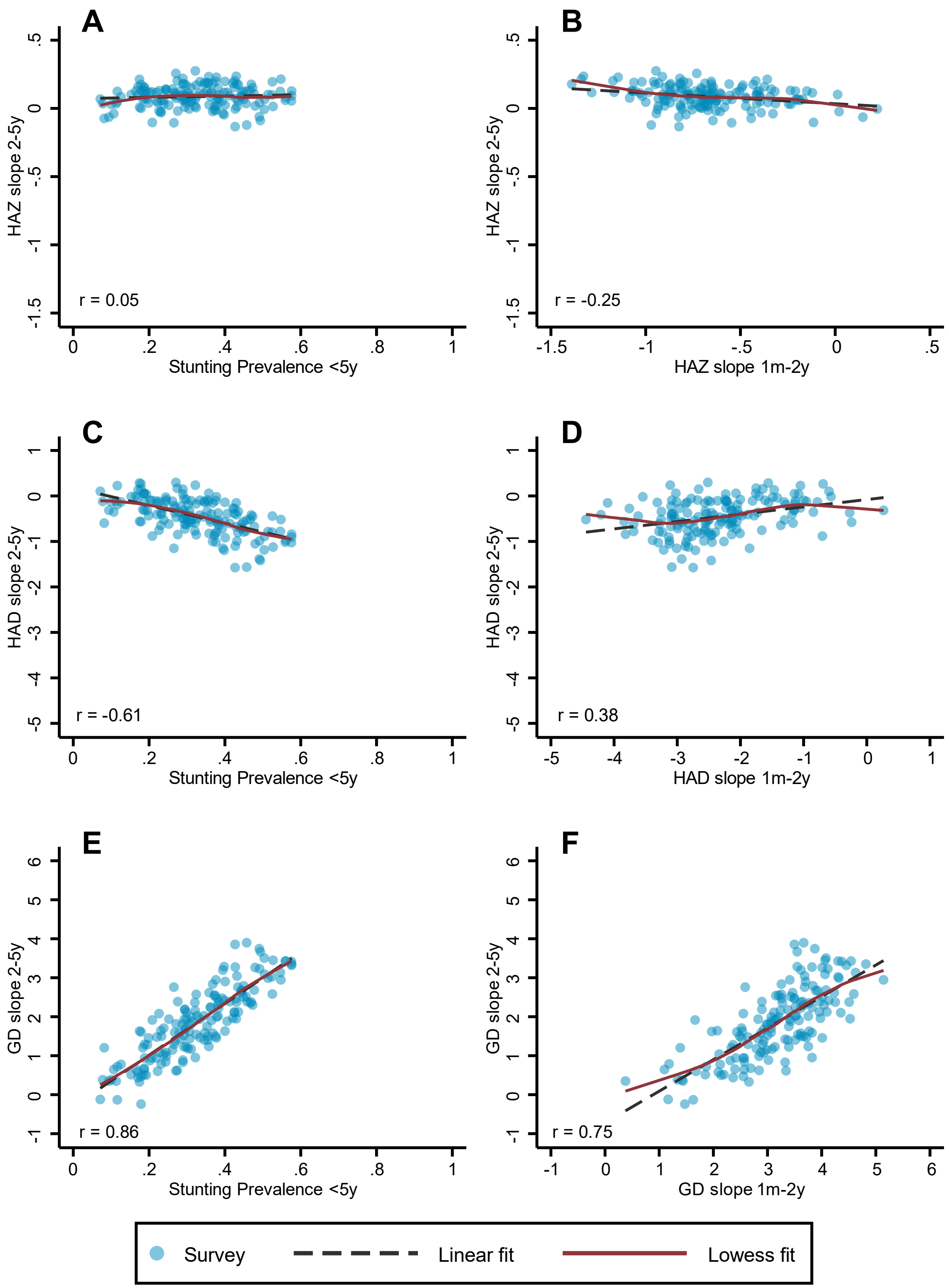

Supplement: S1 Fig — Selected metric-metric relationships shown here demonstrate the relationships of slope metrics between the 1m-2y and 2-5y age ranges (both shown in units of HAZ) and the relationships of the slopes in the 2-5y range with stunting prevalence <5y (shown as a proportion). Each blue circle represents one Demographic and Health Survey (N = 156. Abbreviations: Growth Delay (GD), Height-for-age difference (HAD), Height-for-age z score (HAZ), Month (m), year (y). (PDF) [file pgph.0001766.s001.pdf]
