# Supplementary material for: Complementary and alternative metrics for tracking population-level trends in child linear growth
Source: PLOS Glob Public Health. 2023 Apr 17;3(4):e0001766. doi: 10.1371/journal.pgph.0001766 (PMC10109512; doi:10.1371/journal.pgph.0001766)

**S2 Fig. Ranking of countries by metric for the 10 countries with the lowest and 10 countries with the highest stunting prevalence.** Panel A, 10 countries with the lowest stunting prevalence. Panel B, 10 countries with the highest stunting prevalence. The most recent survey for each country was used (N=63). Surveys were ranked based on the estimated metric value. Larger values of <5y mean HAZ, <5y p25 HAZ, 2-5y mean HAZ, 2-5y p25 HAZ, predicted HAZ at 2y, <5y SITAR-IP, predicted HAZ at 5y, 1m-2y HAD slope, predicted HAZ at birth, 2-5y HAZ slope, and 2-5y HAD slope indicate less growth faltering and are ranked closer to 1. Larger values of <5y stunting, 2-5y stunting, 2-5y GD slope, and 1m-2y GD slope indicate greater growth faltering and are ranked further from 1. Candidate alternative metrics have a ≥|0.95| Spearman correlation with stunting <5y and an absolute Spearman correlation with under 5y mortality, gross domestic product, and the proportion of women with secondary education or higher that is the same or higher than the correlation of stunting <5y with these 3 population health indicators. Candidate complementary metrics have a ≥|0.95| Spearman correlation with stunting <5y and are moderately correlated with the 3 population health indicators. Other metrics are those which did not meet the criteria for candidate alternative or complementary. Abbreviations: Growth Delay (GD), Height-for-age difference (HAD), Height-for-age z score (HAZ), Month (m), year (y).


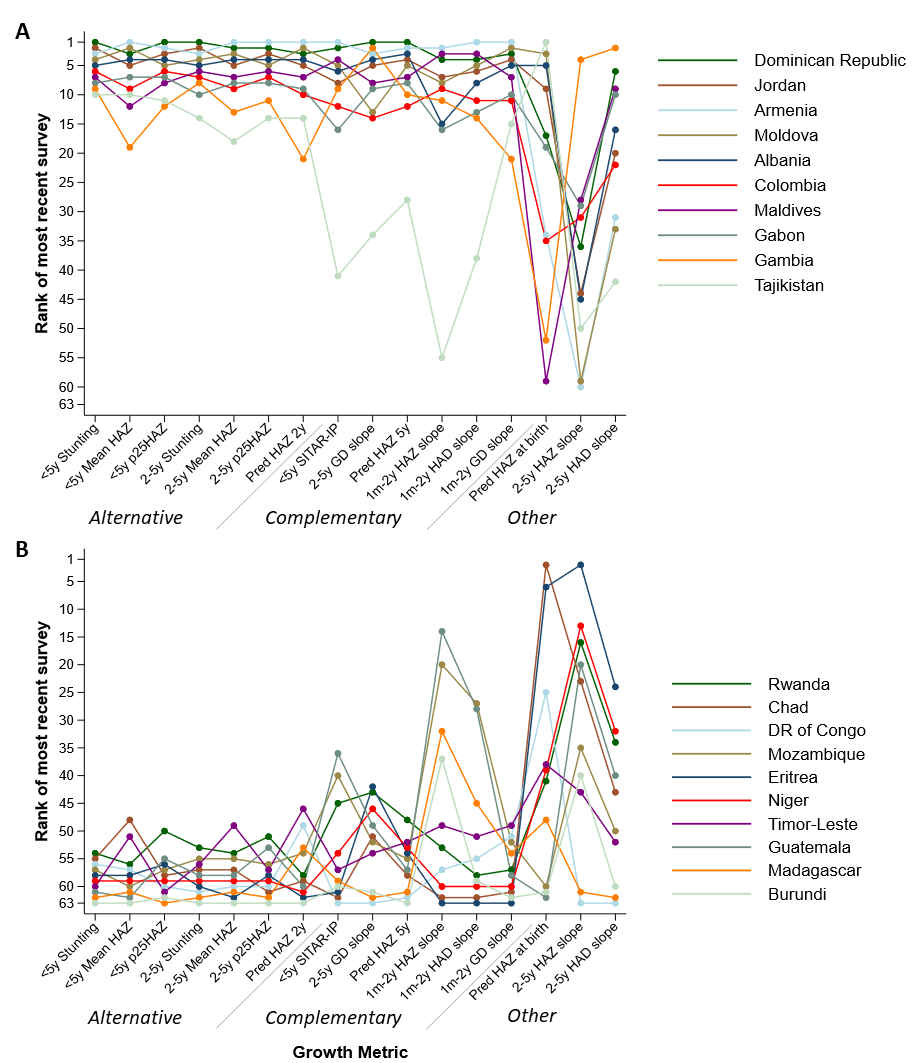

Supplement: S2 Fig — Panel A, 10 countries with the lowest stunting prevalence. Panel B, 10 countries with the highest stunting prevalence. The most recent survey for each country was used (N = 63). Surveys were ranked based on the estimated metric value. Larger values of <5y mean HAZ, <5y p25 HAZ, 2-5y mean HAZ, 2-5y p25 HAZ, predicted HAZ at 2y, <5y SITAR-IP, predicted HAZ at 5y, 1m-2y HAD slope, predicted HAZ at birth, 2-5y HAZ slope, and 2-5y HAD slope indicate less growth faltering and are ranked closer to 1. Larger values of <5y stunting, 2-5y stunting, 2-5y GD slope, and 1m-2y GD slope indicate greater growth faltering and are ranked further from 1. Candidate alternative metrics have a ≥|0.95| Spearman correlation with stunting <5y and an absolute Spearman correlation with under 5y mortality, gross domestic product, and the proportion of women with secondary education or higher that is the same or higher than the correlation of stunting <5y with these 3 population health indicators. Candidate complementary metrics have a ≥|0.95| Spearman correlation with stunting <5y and are moderately correlated with the 3 population health indicators. Other metrics are those which did not meet the criteria for candidate alternative or complementary. Abbreviations: Growth Delay (GD), Height-for-age difference (HAD), Height-for-age z score (HAZ), Month (m), year (y). (DOCX) [file pgph.0001766.s002.docx]
