# Supplementary material for: Complementary and alternative metrics for tracking population-level trends in child linear growth
Source: PLOS Glob Public Health. 2023 Apr 17;3(4):e0001766. doi: 10.1371/journal.pgph.0001766 (PMC10109512; doi:10.1371/journal.pgph.0001766)

**S3 Fig. Time trends of correlations of candidate linear growth metrics with population indicators.** Each analysis included one selected Demographic and Health survey from each country (N=63) that was either the ‘earliest or only’ survey, the survey closest to the midpoint year (2010), or the ‘more recent or only’ survey. Under 5y mortality rate defined as the number of deaths before five years of age per 1000 live births. GDP defined per capita adjusted for purchasing power parity in 2017 in constant international dollars. Abbreviations: Growth Delay (GD), Height-for-age difference (HAD), Height-for-age z score (HAZ), Month (m), year (y)


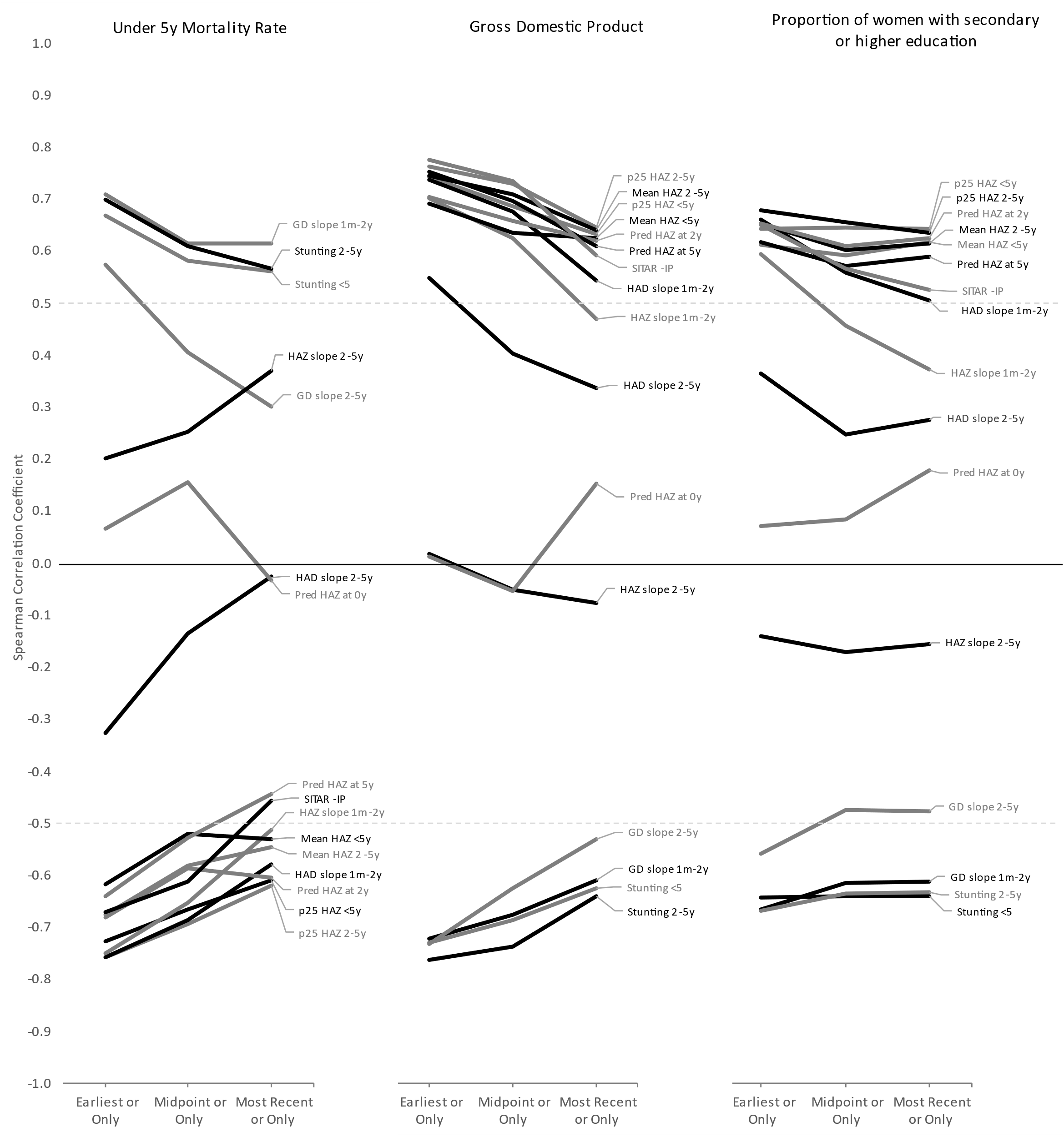

Supplement: S3 Fig — Each analysis included one selected Demographic and Health survey from each country (N = 63) that was either the ‘earliest or only’ survey, the survey closest to the midpoint year (2010), or the ‘more recent or only’ survey. Under 5y mortality rate defined as the number of deaths before five years of age per 1000 live births. GDP defined per capita adjusted for purchasing power parity in 2017 in constant international dollars. Abbreviations: Growth Delay (GD), Height-for-age difference (HAD), Height-for-age z score (HAZ), Month (m), year (y). (DOCX) [file pgph.0001766.s003.docx]
