# Supplementary material for: Complementary and alternative metrics for tracking population-level trends in child linear growth
Source: PLOS Glob Public Health. 2023 Apr 17;3(4):e0001766. doi: 10.1371/journal.pgph.0001766 (PMC10109512; doi:10.1371/journal.pgph.0001766)
Supplement: S1 Table — (PDF) [file pgph.0001766.s004.pdf]

S1 Table. Demographic and Health Surveys included in analyses

#	Country	Number of surveys included	Earliest survey	Midpoint survey[1]	Most recent survey	Mean HAZ under 5 years of age for most recent survey
1	Albania	2	2009	2009	2017	-0.33
2	Angola	1	2015	2015	2015	-1.53
3	Armenia	3	2005	2010	2016	-0.14
4	Azerbaijan	1	2006	2006	2006	-1.11
5	Bangladesh	5	2004	2007	2017	-1.37
6	Benin	3	2001	2006	2017	-1.45
7	Bolivia	2	2003	2008	2008	-1.23
8	Burkina Faso	2	2003	2010	2010	-1.39
9	Burundi	2	2010	2010	2016	-2.18
10	Cambodia	4	2000	2010	2014	-1.41
11	Cameroon	3	2004	2011	2018	-1.12
12	Chad	1	2015	2015	2015	-1.50
13	Colombia	1	2010	2010	2010	-0.81
14	Comoros	1	2012	2012	2012	-1.15
15	Congo	2	2005	2011	2011	-1.02
16	Democratic Republic of the Congo	2	2007	2007	2013	-1.60
17	Côte d'Ivoire	1	2012	2012	2012	-1.23
18	Dominican Republic	3	2002	2007	2013	-0.30
19	Egypt	5	2000	2008	2014	-0.53
20	Eritrea	1	2002	2002	2002	-1.65
21	Ethiopia	5	2000	2011	2019	-1.51
22	Gabon	1	2012	2012	2012	-0.70
23	Gambia	2	2013	2013	2020	-1.04
24	Ghana	2	2008	2008	2014	-0.93
25	Guatemala	1	2015	2015	2015	-1.90
26	Guinea	2	2012	2012	2018	-1.05
27	Guyana	1	2009	2009	2009	-0.88
28	Haiti	4	2000	2006	2017	-0.96
29	Honduras	2	2006	2006	2012	-1.11
30	India	2	2006	2006	2015	-1.42
31	Jordan	3	2002	2009	2012	-0.40
32	Kenya	3	2003	2009	2014	-1.14
33	Kyrgyzstan	1	2012	2012	2012	-0.81
34	Lesotho	2	2009	2009	2014	-1.48
35	Liberia	3	2007	2007	2019	-1.33
36	Madagascar	2	2004	2009	2009	-1.77

#	Country	Number of surveys included	Earliest survey	Midpoint survey[1]	Most recent survey	Mean HAZ under 5 years of age for most recent survey
37	Malawi	4	2000	2010	2015	-1.55
38	Maldives	2	2009	2009	2017	-0.86
39	Mali	4	2001	2006	2018	-1.10
40	Moldova	1	2005	2005	2005	-0.24
41	Morocco	1	2003	2003	2003	-0.83
42	Mozambique	2	2003	2011	2011	-1.69
43	Myanmar	1	2016	2016	2016	-1.32
44	Namibia	3	2000	2007	2007	-0.98
45	Nepal	4	2001	2011	2016	-1.52
46	Nicaragua	1	2001	2001	2001	-1.13
47	Niger	2	2006	2012	2012	-1.68
48	Nigeria	4	2003	2008	2018	-1.52
49	Pakistan	2	2012	2012	2018	-1.56
50	Peru	6	2005	2009	2012	-1.05
51	Rwanda	4	2000	2010	2015	-1.58
52	Sao Tome and Principe	1	2008	2008	2008	-1.17
53	Senegal	9	2005	2010	2019	-0.96
54	Sierra Leone	3	2008	2008	2019	-1.27
55	South Africa	1	2016	2016	2016	-1.15
56	Swaziland	1	2006	2006	2006	-1.24
57	Tajikistan	2	2012	2012	2017	-0.82
58	Tanzania	3	2004	2010	2015	-1.46
59	Timor-Leste	2	2009	2009	2016	-1.52
60	Togo	1	2014	2014	2014	-1.23
61	Uganda	4	2000	2011	2016	-1.20
62	Zambia	3	2002	2007	2018	-1.46
63	Zimbabwe	4	2005	2010	2015	-1.24

[1]Midpoint survey was defined as the survey closest to 2010.
