# Supplementary material for: Complementary and alternative metrics for tracking population-level trends in child linear growth
Source: PLOS Glob Public Health. 2023 Apr 17;3(4):e0001766. doi: 10.1371/journal.pgph.0001766 (PMC10109512; doi:10.1371/journal.pgph.0001766)
Supplement: S2 Table — Values shown are Spearman’s correlation coefficient (95% confidence interval), n = 156 surveys. These are the same data shown in Table 3 but the 95% confidence intervals are additionally shown here. Grey shaded cells have an absolute correlation coefficient of ≥ 0.95. Abbreviations: 25th percentile (p25), Growth delay (GD), Height-for-age difference (HAD), Height-for-age z score (HAZ), Month (m), Predicted (Pred), Prevalence (Prev.), Super Imposition by Translation and Rotation Intensity Parameter (SITAR-IP), year (y). (PDF) [file pgph.0001766.s005.pdf]

S2 Table. Pairwise correlations between linear growth metrics. Values shown are Spearman's correlation coefficient (95% confidence interval), n = 156 surveys. These are the same data shown in Table 3 but the 95% confidence intervals are additionally shown here. Grey shaded cells have an absolute correlation coefficient of ≥ 0.95. Abbreviations: 25th percentile (p25), Growth delay (GD), Height-for-age difference (HAD), Height-for-age z score (HAZ), Month (m), Predicted (Pred), Prevalence (Prev.), Super Imposition by Translation and Rotation Intensity Parameter (SITAR-IP), year (y)

		**<5 years**					**2-5 years**							**1 month – 2 years**			
		Stunting Prev.	Mean HAZ	p25 HAZ	SITAR-IP	Pred. HAZ 0y	Stunting Prev.	Mean HAZ	p25 HAZ	HAZ slope	HAD slope	GD slope	Pred HAZ 5y	HAZ slope	HAD slope	GD slope	Pred HAZ 2y
**<5 years**	Stunting Prev.		-0.97 (-0.98, -0.96)	-0.99 (-0.99, -0.98)	-0.88 (-0.91, -0.84)	-0.32 (-0.46, -0.17)	0.99 (0.98, 0.99)	-0.97 (-0.98, -0.96)	-0.97 (-0.98, -0.96)	0.05 (-0.11, 0.21)	-0.62 (-0.70, -0.51)	0.86 (0.81, 0.90)	-0.94 (-0.96, -0.92)	-0.69 (-0.76, -0.60)	-0.83 (-0.87, -0.77)	0.94 (0.91, 0.95)	-0.95 (-0.96, -0.93)
	Mean HAZ	-0.97 (-0.98, -0.96)		0.94(0.92, 0.96)	0.86 (0.81, 0.90)	0.42 (0.28, 0.54)	-0.96 (-0.97, -0.95)	0.98 (0.97, 0.98)	0.93 (0.90, 0.95)	-0.08 (-0.24, 0.08)	0.60 (0.49, 0.69)	-0.86 (-0.89, -0.81)	0.95 (0.93, 0.96)	0.64 (0.53, 0.72)	0.80 (0.73, 0.85)	-0.94 (-0.95, -0.91)	0.96 (0.95, 0.97)
	p25 HAZ	-0.99 (-0.99, -0.98)	0.94 (0.92, 0.96)		0.88 (0.83, 0.91)	0.27 (0.11, 0.41)	-0.98 (-0.98, -0.97)	0.95 (0.94, 0.97)	0.99 (0.98, 0.99)	-0.07 (-0.22, 0.09)	0.59 (0.48, 0.69)	-0.84 (-0.88, -0.79)	0.93 (0.90, 0.95)	0.71 (0.63, 0.78)	0.84 (0.79, 0.88)	-0.93 (-0.95, -0.90)	0.93 (0.91, 0.95)
	SITAR IP	-0.88 (-0.91, -0.84)	0.86 (0.81, 0.90)	0.88 (0.83, 0.91)		0.02 (-0.14, 0.18)	-0.93 (-0.95, -0.90)	0.93 (0.91, 0.95)	0.92 (0.89, 0.94)	0.13 (-0.03, 0.28)	0.73 (0.65, 0.80)	-0.92 (-0.94, -0.89)	0.95 (0.93, 0.96)	0.81 (0.75, 0.86)	0.87 (0.83, 0.91)	-0.88 (-0.91, -0.84)	0.86 (0.81, 0.89)
	Pred. HAZ 0y	-0.32 (-0.46, -0.17)	0.42 (0.28, 0.54)	0.27 (0.11, 0.41)	0.02 (-0.14, 0.18)		-0.24 (-0.38, -0.09)	0.26 (0.11, 0.40)	0.18 (0.02, 0.33)	<0.01 (-0.16, 0.15)	0.19 (0.04, 0.34)	-0.25 (-0.39, -0.10)	0.26 (0.11, 0.40)	-0.35 (-0.48, -0.21)	-0.12 (-0.28, 0.03)	-0.16 (-0.31, -0.01)	0.25 (0.10, 0.40)
**2-5 years**	Stunting Prev.	0.99(0.98, 0.99)	-0.96(-0.97, - 0.95)	-0.98 (-0.98, -0.97)	-0.93 (-0.95, -0.90)	-0.24 (-0.38, -0.09)		-0.99 (-0.99, -0.98)	-0.98 (-0.99, -0.98)	0.03 (-0.13, 0.18)	-0.65 (-0.73, -0.55)	0.89 (0.85, 0.92)	-0.97 (-0.98, -0.96)	-0.74 (-0.80, -0.66)	-0.87 (-0.90, -0.82)	0.95 (0.93, 0.96)	-0.95(-0.97, -0.94)
	Mean HAZ	-0.97 (-0.98, -0.96)	0.98 (0.97, 0.98)	0.95 (0.94, 0.97)	0.93 (0.91, 0.95)	0.26 (0.11, 0.40)	-0.99 (-0.99, -0.98)		0.96 (0.95, 0.97)	-0.04 (-0.20, 0.11)	0.64 (0.54, 0.72)	-0.89 (-0.92, -0.85)	0.97 (0.96, 0.98)	0.74 (0.66, 0.81)	0.87 (0.83, 0.91)	-0.96 (-0.97, -0.95)	0.97 (0.96, 0.98)
	p25 HAZ	-0.97 (-0.98, -0.96)	0.93 (0.90, 0.95)	0.99 (0.98, 0.99)	0.92 (0.89, 0.94)	0.18 (0.02, 0.33)	-0.98 (-0.99, -0.98)	0.96 (0.95, 0.97)		-0.04 (-0.20, 0.12)	0.62 (0.52, 0.71)	-0.87 (-0.90, -0.82)	0.95 (0.93, 0.96)	0.76 (0.69, 0.82)	0.87 (0.83, 0.91)	-0.94 (-0.95, -0.91)	0.93 (0.91, 0.95)
	HAZ slope	0.05 (-0.11, 0.21)	-0.08 (-0.24, 0.08)	-0.07 (-0.22, 0.09)	0.13 (-0.03, 0.28)	<0.01 (-0.16, 0.15)	0.03 (-0.13, 0.18)	-0.04 (-0.20, 0.11)	-0.04 (-0.20, 0.12)		0.71 (0.62, 0.78)	-0.37 (-0.50, -0.23)	0.15 (0.00, 0.30)	-0.25 (-0.39, -0.10)	-0.27 (-0.41, -0.12)	0.27 (0.12, 0.41)	-0.26 (-0.40, -0.11)
	HAD slope	-0.62 (-0.70, -0.51)	0.60 (0.49, 0.69)	0.59 (0.48, 0.69)	0.73 (0.65, 0.80)	0.19 (0.04, 0.34)	-0.65 (-0.73, -0.55)	0.64 (0.54, 0.72)	0.62 (0.52, 0.71)	0.71 (0.62, 0.78)		-0.91 (-0.93, -0.88)	0.79 (0.72, 0.84)	0.31 (0.16, 0.45)	0.38 (0.24, 0.51)	-0.44(-0.56, - 0.31)	0.45 (0.32, 0.57)
	GD slope	0.86 (0.81, 0.90)	-0.86 (-0.89, -0.81)	-0.84 (-0.88, -0.79)	-0.92 (-0.94, -0.89)	-0.25 (-0.39, -0.10)	0.89 (0.85, 0.92)	-0.89 (-0.92, -0.85)	-0.87 (-0.90, -0.82)	-0.37 (-0.50, -0.23)	-0.91 (-0.93, -0.88)		-0.97 (-0.98, -0.96)	-0.57 (-0.67, -0.45)	-0.67 (-0.75, -0.58)	0.75 (0.68, 0.81)	-0.76 (-0.82, -0.69)
	Pred HAZ 5y	-0.94 (-0.96, -0.92)	0.95 (0.93, 0.96)	0.93 (0.90, 0.95)	0.95 (0.93, 0.96)	0.26 (0.11, 0.40)	-0.97 (-0.98, -0.96)	0.97 (0.96, 0.98)	0.95 (0.93, 0.96)	0.15 (0.00, 0.30)	0.79 (0.72, 0.84)	-0.97 (-0.98, -0.96)		0.68 (0.58, 0.76)	0.80 (0.74, 0.85)	-0.89 (-0.92, -0.85)	0.89 (0.86, 0.92)
**1 month - 2 years**	HAZ slope	-0.69 (-0.76, -0.60)	0.64 (0.53, 0.72)	0.71 (0.63, 0.78)	0.81 (0.75, 0.86)	-0.35 (-0.48, -0.21)	-0.74 (-0.80, -0.66)	0.74 (0.66, 0.81)	0.76 (0.69, 0.82)	-0.25 (-0.39, -0.10)	0.31 (0.16, 0.45)	-0.57 (-0.67, -0.45)	0.68 (0.58, 0.76)		0.96 (0.95, 0.97)	-0.83 (-0.87, -0.77)	0.77 (0.70, 0.83)
	HAD slope	-0.83 (-0.87, -0.77)	0.80 (0.73, 0.85)	0.84 (0.79, 0.88)	0.87 (0.83, 0.91)	-0.12 (-0.28, 0.03)	-0.87 (-0.90, -0.82)	0.87 (0.83, 0.91)	0.87 (0.83, 0.91)	-0.27 (-0.41, -0.12)	0.38 (0.24, 0.51)	-0.67 (-0.75, -0.58)	0.80 (0.74, 0.85)	0.96 (0.95, 0.97)		-0.94 (-0.96, -0.92)	0.91 (0.87, 0.93)
	GD slope	0.94 (0.91, 0.95)	-0.94 (-0.95, -0.91)	-0.93 (-0.95, -0.90)	-0.88 (-0.91, -0.84)	-0.16 (-0.31, -0.01)	0.95 (0.93, 0.96)	-0.96 (-0.97, -0.95)	-0.94 (-0.95, -0.91)	0.27 (0.12, 0.41)	-0.44 (-0.56, -0.31)	0.75 (0.68, 0.81)	-0.89 (-0.92, -0.85)	-0.83 (-0.87, -0.77)	-0.94 (-0.96, -0.92)		-0.99 (-1.00, -0.99)
	Pred HAZ 2y	-0.95 (-0.96, -0.93)	0.96 (0.95, 0.97)	0.93 (0.91, 0.95)	0.86 (0.81, 0.89)	0.25 (0.10, 0.40)	-0.95 (-0.97, -0.94)	0.97 (0.96, 0.98)	0.93 (0.91, 0.95)	-0.26 (-0.40, -0.11)	0.45 (0.32, 0.57)	-0.76 (-0.82, -0.69)	0.89 (0.86, 0.92)	0.77 (0.70, 0.83)	0.91 (0.87, 0.93)	-0.99 (-1.00, -0.99)	
		Stunting Prev.	Mean HAZ	p25 HAZ	SITAR-IP	Pred. HAZ 0y	Stunting Prev.	Mean HAZ	p25 HAZ	HAZ slope	HAD slope	GD slope	Pred HAZ 5y	HAZ slope	HAD slope	GD slope	Pred HAZ 2y
		**<5 years**					**2-5 years**							**1 month – 2 years**			
