# Supplementary material for: Complementary and alternative metrics for tracking population-level trends in child linear growth
Source: PLOS Glob Public Health. 2023 Apr 17;3(4):e0001766. doi: 10.1371/journal.pgph.0001766 (PMC10109512; doi:10.1371/journal.pgph.0001766)
Supplement: S3 Table — Values shown are Spearman’s correlation coefficient (95% confidence interval). Grey shaded cells are considered as candidate alternative metrics to stunting prevalence (absolute Spearman correlation coefficient with under 5y stunting prevalence is ≥ 0.95). Under 5y mortality rate defined as the number of deaths before five years of age per 1000 live births. GDP defined per capita adjusted for purchasing power parity in 2017 in constant international dollars. Abbreviations: 25th percentile (p25), Growth delay (GD), Height-for-age difference (HAD), Height-for-age z score (HAZ), Month (m), Predicted (Pred), Prevalence (Prev.), Super Imposition by Translation and Rotation Intensity Parameter (SITAR-IP), year (y). (PDF) [file pgph.0001766.s006.pdf]

S3 Table. Correlations between linear growth metrics and population indicators (N = 156 Demographic and Health Surveys). Values shown are Spearman's correlation coefficient (95% confidence interval). Grey shaded cells are considered as candidate alternative metrics to stunting prevalence (absolute Spearman correlation coefficient with under 5y stunting prevalence is ≥ 0.95). Under 5y mortality rate defined as the number of deaths before five years of age per 1000 live births. GDP defined per capita adjusted for purchasing power parity in 2017 in constant international dollars. Abbreviations: 25th percentile (p25), Growth delay (GD), Height-for-age difference (HAD), Height-for-age z score (HAZ), Month (m), Predicted (Pred), Prevalence (Prev.), Super Imposition by Translation and Rotation Intensity Parameter (SITAR-IP), year (y)

		Population Indicator		
**Age interval**	**Growth Metric**	**Under 5y Mortality Rate**	**Gross Domestic Product**	**Proportion of Women with Secondary or Higher Education**
<5 years	Stunting Preval.	0.65 (0.54, 0.73)	-0.71 (-0.78, -0.62)	-0.59 (-0.68, -0.48)
	Mean HAZ	-0.58 (-0.68, -0.46)	0.69 (0.60, 0.76)	0.56 (0.44, 0.66)
	p25 HAZ	-0.71 (-0.78, -0.62)	0.70 (0.61, 0.77)	0.59 (0.48, 0.69)
	SITAR IP	-0.62 (-0.71, -0.51)	0.70 (0.62, 0.78)	0.56 (0.45, 0.66)
	Pred. HAZ 0y	0.08 (-0.07, 0.24)	0.07 (-0.09, 0.22)	0.04 (-0.12, 0.19)
2 – 5 years	Stunting Preval.	0.67 (0.57, 0.75)	-0.74 (-0.80, -0.66)	-0.61 (-0.70, -0.50)
	Mean HAZ	-0.64 (-0.72, -0.54)	0.73 (0.65, 0.80)	0.60 (0.49, 0.69)
	p25 HAZ	-0.73 (-0.80, -0.65)	0.73 (0.65, 0.80)	0.62 (0.51, 0.71)
	HAZ slope	0.27 (0.12, 0.41)	-0.08 (-0.24, 0.07)	-0.20 (-0.35, -0.05)
	HAD slope	-0.23 (-0.37, -0.08)	0.43 (0.30, 0.55)	0.27 (0.12, 0.41)
	GD slope	0.48 (0.35, 0.59)	-0.64 (-0.72, -0.53)	-0.48 (-0.59, -0.34)
	Pred HAZ 5y	-0.58 (-0.68, -0.47)	0.70 (0.62, 0.78)	0.56 (0.44, 0.66)
1 month – 2 years	HAZ slope	-0.69 (-0.77, -0.60)	0.64 (0.53, 0.72)	0.54 (0.42, 0.64)
	HAD slope	-0.72 (-0.79, -0.64)	0.70 (0.61, 0.77)	0.60 (0.49, 0.70)
	GD slope	0.69 (0.60, 0.77)	-0.72 (-0.79, -0.64)	-0.63 (-0.72, -0.52)
	Pred HAZ 2y	-0.67 (-0.74, -0.57)	0.71 (0.62, 0.78)	0.62 (0.51, 0.70)
