# Supplementary material for: Complementary and alternative metrics for tracking population-level trends in child linear growth
Source: PLOS Glob Public Health. 2023 Apr 17;3(4):e0001766. doi: 10.1371/journal.pgph.0001766 (PMC10109512; doi:10.1371/journal.pgph.0001766)
Supplement: S1 File — (DOCX) [file pgph.0001766.s007.docx]

**DATA ACCESS INSTRUCTIONS**

*Version 2023-02-16*

**Complementary and alternative metrics for tracking population-level trends in child linear growth**

Ashley M. Aimone, Diego G. Bassani, Huma Qamar, Alison Dasiewicz, Nandita Perumal, Sorrel M.L. Namaste, Devanshi Shah, Daniel E. Roth^*^

^*^ Corresponding author:

E-mail: daniel.roth@sickkids.ca (DER)

Contents

[1 Accessing DHS Datasets 1](#_Toc127428879)

[2 Accessing World Bank Datasets 6](#_Toc127428880)

[2.1 Under-5 mortality 6](#_Toc127428881)

[2.2 Gross Domestic Product 6](#_Toc127428882)

# Accessing DHS Datasets

The Demographic and Health Surveys (DHS) datasets that were used in this research are publicly available to use for research purposes. They can be accessed by the following steps:

1. Register as a new user by navigating to the “Data” page of the DHS website (<https://dhsprogram.com/Data/>) and select the link “Register for Dataset Access”.
2. Fill out the New User Registration Form and continue to the next page.
3. Enter a project title, any co-researchers, and a description of the proposed research project for which the datasets will be used. The variables used in this research are provided in a table starting on page 5 of this document. Continue to the next page.
4. Within each region of the drop-down menu, select the countries displayed in the table below by checking the corresponding “Survey” checkbox.
5. Once all countries in the table below have been selected, click “Submit Dataset Request Now”. DHS Program staff will review the request and send access information to the email address provided during registration.
6. Once approved for access, log in by selecting the “Login” link at the top of the DHS website (<https://dhsprogram.com/>).
7. Select the project title from the dropdown.
8. Select the countries you wish to download data for individually using the dropdown menu, or download multiple datasets at once by selecting the “Download manager”.
9. The datasets used in this research are all of type “Household Member Recode”, denoted by “PR” following the two-character country code in the file name. A list of all datasets used in the research is provided in the table below.

An instructional video demonstrating the above steps is found on the DHS website at the following link: <https://dhsprogram.com/data/Access-Instructions.cfm>.

DHS datasets used in this study:

| **Country** | **Year** | **Dataset** |
| --- | --- | --- |
| Albania | 2009 | ALPR50 |
| Albania | 2017 | ALPR71 |
| Angola | 2015 | AOPR71 |
| Armenia | 2005 | AMPR54 |
| Armenia | 2010 | AMPR61 |
| Armenia | 2016 | AMPR71 |
| Azerbaijan | 2006 | AZPR52 |
| Bangladesh | 2004 | BDPR4j |
| Bangladesh | 2007 | BDPR51 |
| Bangladesh | 2011 | BDPR61 |
| Bangladesh | 2014 | BDPR72 |
| Bangladesh | 2017 | BDPR7R |
| Benin | 2001 | BJPR41 |
| Benin | 2006 | BJPR51 |
| Benin | 2017 | BJPR71 |
| Bolivia | 2003 | BOPR41 |
| Bolivia | 2008 | BOPR51 |
| Burkina Faso | 2003 | BFPR44 |
| Burkina Faso | 2010 | BFPR62 |
| Burundi | 2010 | BUPR61 |
| Burundi | 2016 | BUPR70 |
| Cambodia | 2000 | KHPR42 |
| Cambodia | 2005 | KHPR51 |
| Cambodia | 2010 | KHPR61 |
| Cambodia | 2014 | KHPR73 |
| Cameroon | 2004 | CMPR45 |
| Cameroon | 2011 | CMPR61 |
| Cameroon | 2018 | CMPR71 |
| Chad | 2015 | TDPR71 |
| Colombia | 2010 | COPR61 |
| Comoros | 2012 | KMPR61 |
| Congo | 2005 | CGPR51 |
| Congo | 2011 | CGPR60 |
| Congo Democratic Republic | 2007 | CDPR50 |
| Congo Democratic Republic | 2013 | CDPR61 |
| Cote d'Ivoire | 2012 | CIPR62 |
| Dominican Republic | 2002 | DRPR4a |
| Dominican Republic | 2007 | DRPR52 |
| Dominican Republic | 2013 | DRPR61 |
| Egypt | 2000 | EGPR42 |
| Egypt | 2003 | EGPR4a |
| Egypt | 2005 | EGPR51 |
| Egypt | 2008 | EGPR5a |
| Egypt | 2014 | EGPR61 |
| Eritrea | 2002 | ERPR41 |
| Ethiopia | 2000 | ETPR41 |
| Ethiopia | 2005 | ETPR51 |
| Ethiopia | 2011 | ETPR61 |
| Ethiopia | 2016 | ETPR70 |
| Ethiopia | 2019 | ETPR81 |
| Gabon | 2012 | GAPR60 |
| Gambia | 2013 | GMPR60 |
| Gambia | 2020 | GMPR81 |
| Ghana | 2008 | GHPR5a |
| Ghana | 2014 | GHPR72 |
| Guatemala | 2015 | GUPR71 |
| Guinea | 2012 | GNPR62 |
| Guinea | 2018 | GNPR71 |
| Guyana | 2009 | GYPR5I |
| Haiti | 2000 | HTPR42 |
| Haiti | 2006 | HTPR52 |
| Haiti | 2012 | HTPR61 |
| Haiti | 2017 | HTPR70 |
| Honduras | 2006 | HNPR52 |
| Honduras | 2012 | HNPR62 |
| India | 2006 | IAPR52 |
| India | 2015 | IAPR73 |
| Jordan | 2002 | JOPR42 |
| Jordan | 2009 | JOPR61 |
| Jordan | 2012 | JOPR6C |
| Kenya | 2003 | KEPR42 |
| Kenya | 2009 | KEPR52 |
| Kenya | 2014 | KEPR71 |
| Kyrgyz Republic | 2012 | KYPR61 |
| Lesotho | 2009 | LSPR61 |
| Lesotho | 2014 | LSPR71 |
| Liberia | 2007 | LBPR51 |
| Liberia | 2013 | LBPR6a |
| Liberia | 2019 | LBPR7A |
| Madagascar | 2004 | MDPR42 |
| Madagascar | 2009 | MDPR51 |
| Malawi | 2000 | MWPR41 |
| Malawi | 2004 | MWPR4e |
| Malawi | 2010 | MWPR61 |
| Malawi | 2015 | MWPR7H |
| Maldives | 2009 | MVPR52 |
| Maldives | 2017 | MVPR71 |
| Mali | 2001 | MLPR41 |
| Mali | 2006 | MLPR53 |
| Mali | 2012 | MLPR6H |
| Mali | 2018 | MLPR7A |
| Moldova | 2005 | MBPR53 |
| Morocco | 2003 | MAPR43 |
| Mozambique | 2003 | MZPR41 |
| Mozambique | 2011 | MZPR62 |
| Myanmar | 2016 | MMPR71 |
| Namibia | 2000 | NMPR41 |
| Namibia | 2007 | NMPR52 |
| Namibia | 2013 | NMPR61 |
| Nepal | 2001 | NPPR41 |
| Nepal | 2006 | NPPR51 |
| Nepal | 2011 | NPPR60 |
| Nepal | 2016 | NPPR7H |
| Nicaragua | 2001 | NCPR41 |
| Niger | 2006 | NIPR51 |
| Niger | 2012 | NIPR61 |
| Nigeria | 2003 | NGPR4c |
| Nigeria | 2008 | NGPR53 |
| Nigeria | 2013 | NGPR6A |
| Nigeria | 2018 | NGPR7A |
| Pakistan | 2012 | PKPR61 |
| Pakistan | 2018 | PKPR71 |
| Peru | 2005 | PEPR51 |
| Peru | 2008 | PEPR51 |
| Peru | 2009 | PEPR51 |
| Peru | 2010 | PEPR61 |
| Peru | 2011 | PEPR6A |
| Peru | 2012 | PEPR6I |
| Rwanda | 2000 | RWPR41 |
| Rwanda | 2005 | RWPR53 |
| Rwanda | 2010 | RWPR61 |
| Rwanda | 2015 | RWPR70 |
| Sao Tome and Principe | 2008 | STPR50 |
| Senegal | 2005 | SNPR4H |
| Senegal | 2010 | SNPR61 |
| Senegal | 2013 | SNPR6d |
| Senegal | 2014 | SNPR70 |
| Senegal | 2015 | SNPR7H |
| Senegal | 2016 | SNPR7I |
| Senegal | 2017 | SNPR7Z |
| Senegal | 2018 | SNPR81 |
| Senegal | 2019 | SNPR8B |
| Sierra Leone | 2008 | SLPR51 |
| Sierra Leone | 2013 | SLPR61 |
| Sierra Leone | 2019 | SLPR7A |
| South Africa | 2016 | ZAPR71 |
| Swaziland | 2006 | SZPR52 |
| Tajikistan | 2012 | TJPR61 |
| Tajikistan | 2017 | TJPR70 |
| Tanzania | 2004 | TZPR4i |
| Tanzania | 2010 | TZPR63 |
| Tanzania | 2015 | TZPR7H |
| Timor-Leste | 2009 | TLPR61 |
| Timor-Leste | 2016 | TLPR71 |
| Togo | 2014 | TGPR61 |
| Uganda | 2000 | UGPR41 |
| Uganda | 2006 | UGPR52 |
| Uganda | 2011 | UGPR60 |
| Uganda | 2016 | UGPR7h |
| Zambia | 2002 | ZMPR43 |
| Zambia | 2007 | ZMPR51 |
| Zambia | 2013 | ZMPR61 |
| Zambia | 2018 | ZMPR71 |
| Zimbabwe | 2005 | ZWPR52 |
| Zimbabwe | 2010 | ZWPR62 |
| Zimbabwe | 2015 | ZWPR71 |

Variables required from DHS datasets.

| **Variable description** | **Variable name** |
| --- | --- |
| Survey weight | hv005 |
| Survey cluster | hv021 or sh021 |
| Survey strata | hv023 or groups of hv024 and hv025 |
| Sex of child | hc27 |
| De facto residence of child | hv103 |
| Child height | hc3 |
| Child measured lying down or standing | hc15 |
| Day of birth of child | hc16 |
| Month of birth of child | hc30 |
| Year of birth of child | hc31 |
| Day of measurement | hc17 |
| Day of measurement | hc18 |
| Year of measurement | hc19 |
| Mother’s highest education level | hc61 |

# Accessing World Bank Datasets

## Under-5 mortality

The under-5 mortality dataset used in this research is readily available online and can be accessed by the following steps:

1. On the World Bank Open Data website (<https://data.worldbank.org/>) select “Indicator” to browse by indicators.
2. Under the “Aid Effectiveness” heading, select “Mortality rate, under-5 (per 1,000 live births)”.
3. On the “Download” panel, select the type of file.

## Gross Domestic Product

The gross domestic product (GDP) dataset used in this research is readily available online and can be accessed by the following steps:

1. On the World Bank Open Data website (<https://data.worldbank.org/>) select “Indicator” to browse by indicators.
2. Under the “Economy & Growth” heading, select “GDP per capita, PPP (constant 2017 international $)”.
3. On the “Download” panel, select the type of file.
